# SUMOylation is a Translatable Target in Hypoxic MNPs Regulating Retinal Vasculopathy

**DOI:** 10.1002/advs.202503505

**Published:** 2025-05-31

**Authors:** Zheng Zhong, Guangyu Liang, Huimin Yu, Jiaqi Li, Ruohong Wang, Xiaohong Ma, Ziqing Zhou, Yin Zhao, Fei Sun, Xufang Sun

**Affiliations:** ^1^ Department of Ophthalmology Tongji Hospital Tongji Medical College Huazhong University of Science and Technology Wuhan 430030 China; ^2^ Experimental Medicine Center Tongji Hospital Tongji Medical College Huazhong University of Science and Technology Wuhan 430000 China; ^3^ Key Laboratory of Otorhinolaryngologic and Ophthalmic Diseases Tongji Hospital Tongji Medical College Huazhong University of Science and Technology Wuhan 430000 China; ^4^ The Center for Biomedical Research NHC Key Laboratory of Respiratory Diseases Tongji Hospital Tongji Medical College Huazhong University of Science and Technology Wuhan 430000 China

**Keywords:** pro‐angiogenic MNPs, retinal vasculopathies, SUMOylation, UBC9

## Abstract

Retinal vasculopathies pose a devastating threat to human health. While anti‐VEGF therapy situates the first‐line treatment for patients, the clinical efficacy is limited by suboptimal response and potential risks raised by long‐term high‐dosage use. Neurovascular unit uncoupling is recognized as a key mechanism contributing to pathological neovascularization, yet how immune components get involved is less appreciated. Here, it is reported that SUMOylation modulates the pro‐angiogenic capacity of macrophage, and inhibition of the SUMO‐conjugating enzyme UBC9 synergizes with anti‐VEGF therapy in preclinical models. Diabetic human retinal mononuclear phagocytes (MNPs) overexpress UBC9. Genetic ablation of UBC9 in MNPs compromises the crosstalk with endothelial cells by reducing *Vegfa* splicing isoforms, including *Vegf120*, *Vegf144*, *Vegf164*, and *Vegf188*. Mechanistically, hypoxia facilitates the SUMOylation of fused in sarcoma (FUS) protein at lysine residues K327 and K502. Mutation of the SUMOylation sites enhances FUS binding to the *Vegfa* 3′‐untranslated region (3′UTR), leading to mRNA destabilization and decreased VEGFA production. Intravitreal administration of anti‐VEGF elevates UBC9 whereas Ubc9 siRNA‐liposomes alleviates retinal vascular leakage and choroidal neovascularization, and a better therapeutic efficacy is yielded when combining with anti‐VEGF therapy. Taken together, this study highlights a novel approach for treating retinal vascular diseases by modulating the MNPs‐endothelial cell interplay.

## Graphical abstract

During ischemic retinopathy/retinal vasculopathy, UBC9‐mediated SUMOylation in retinal macrophages enhances their pro‐angiogenic capacity via hypoxia‐induced SUMOylation of FUS at K327/K502. This modification suppresses FUS binding to the *Vegfa* mRNA 3’UTR, stabilizing transcripts and facilitating VEGFA production. Targeting UBC9 inhibition can serve as a potential therapeutic strategy for retinal vasculopathy, with a combination of anti‐VEGF therapy demonstrating promising synergistic effects.

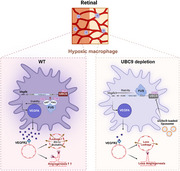

## 1 Introduction

Central vision loss or blurriness occurs in patients with retinal vascular diseases including neovascular age‐related macular degeneration (nAMD), diabetic retinopathy (DR), retinal vein occlusion, and retinopathy of prematurity. In addition to neuroretinal damage, these diseases are characterized by hypoxic microenvironment, enhanced vascular permeability, derailed angiogenesis, and immaturity of neovessels. The upregulation of VEGFA is observed in the vitreous or aqueous fluid of patients with nAMD or DR. In clinical practice, anti‐VEGF therapy improves the prognosis of diabetic edema and nAMD, with real‐world studies corroborating its efficacy^[^ ^1^, ^2^ ^]^ Nevertheless, a significant proportion of patients exhibit incomplete or suboptimal responses, even following intensive treatment regimens.^[^ ^3^ ^]^ Addressing this therapeutic challenge necessitates the development of novel strategies tailored to non‐responsive cases. However, the underlying causal factors driving treatment resistance remain poorly understood.^[^ ^4^ ^]^

Persistent efforts have been made to elucidate the mechanisms underlying neurovascular uncoupling.^[^ ^5^ ^]^ Evidence suggests that pericyte loss and Müller cell activation compromise the integrity of the neurovascular unit, exacerbating retinal dysfunction.^[^ ^6^, ^7^ ^]^ In parallel, mononuclear phagocytes (MNPs), including circulating monocytes, tissue‐resident microglia, and monocyte‐derived macrophages, interact with endothelial cells via the immune‐vascular circuit, which is facilitated by their close anatomical proximity. Cooperatively, microglia/macrophages contribute to retinal homeostasis through modulating vessel diameter^[^ ^8^ ^]^ or the process of angiogenesis.^[^ ^9^ ^]^ In the advanced stages of DR or nAMD, mixed angiogenic factors, including VEGFA, IGF1, and TNF‐α, are secreted from microglia/macrophages to promote pathological angiogenesis. Numerous studies have highlighted the potential to manipulate macrophage/microglia polarization and activation to intervene retinal angiogenesis.^[^ ^10^ ^]^ Therefore, the versatility and plasticity of MNPs render them feasible pharmacological targets for the treatment of retinal vascular diseases.

SUMOylation, a post‐translational modification (PTM) involving the conjugation of small ubiquitin‐like modifiers (SUMO), plays a pivotal role in various pathophysiological processes.^[^ ^11^ ^]^ Emerging evidence has unraveled its significant impact on macrophage/microglia polarization and reprogramming. For instance, repressed SUMOylation promotes the M1 program but hinders IL‐4‐induced M2 phenotype.^[^ ^12^, ^13^ ^]^ SUMOylation of annexin A1 (ANXA1) in microglia has been shown to attenuate inflammatory cytokine production in the oxygen‐glucose deprivation/reperfusion model.^[^ ^14^ ^]^ In the presence of rosiglitazone, SUMOylation of peroxisome proliferator‐activated receptor gamma (PPAR‐γ) in macrophages downregulates the expression of inducible nitric oxide synthase, thereby curbing inflammation.^[^ ^15^ ^]^ Conversely, oxidized low‐density lipoprotein stimulation intensifies atherosclerosis by facilitating macrophage‐foam cell transformation, which is mediated through increased transcription factor EB SUMOylation and consequent lysosomal dysfunction.^[^ ^16^ ^]^ Therefore, the regulatory effects of SUMOylation on MNPs are largely context‐dependent, informing distinct therapeutic strategies under different disease settings.

In the retina, SUMOylation of VEGFR2 in endothelial cells has been shown to mitigate pathological angiogenesis driven by VEGF164 overexpression.^[^ ^17^ ^]^ Controversially, excess SUMOylation is observed in the retina of streptozotocin (STZ)‐induced diabetic mice and overexpression of the deSUMOylation enzyme sentrin specific protease 1(SENP1) ameliorates angiogenesis in vivo.^[^ ^18^ ^]^ Overall, the relevant studies are scarce and primarily concentrated on endothelial cells. We speculated that other cellular components, MNPs in particular, are also affected by SUMOylation machinery. In the present study, we investigated the pro‐angiogenic capacity of microglia/macrophages regulated by UBC9‐mediated SUMOylation in retinal vascular diseases and dissected the in‐depth mechanism. Most critically, we assessed the therapeutic potential of the UBC9‐based intervention strategy by using human samples and preclinical animal models. Our work thus paves a novel avenue for the management of retinal vasculopathy.

## 2 Results

### 2.1 Retinal Vasculopathy is Characterized by Upregulation of UBC9 in MNPs

**Figure 1:**
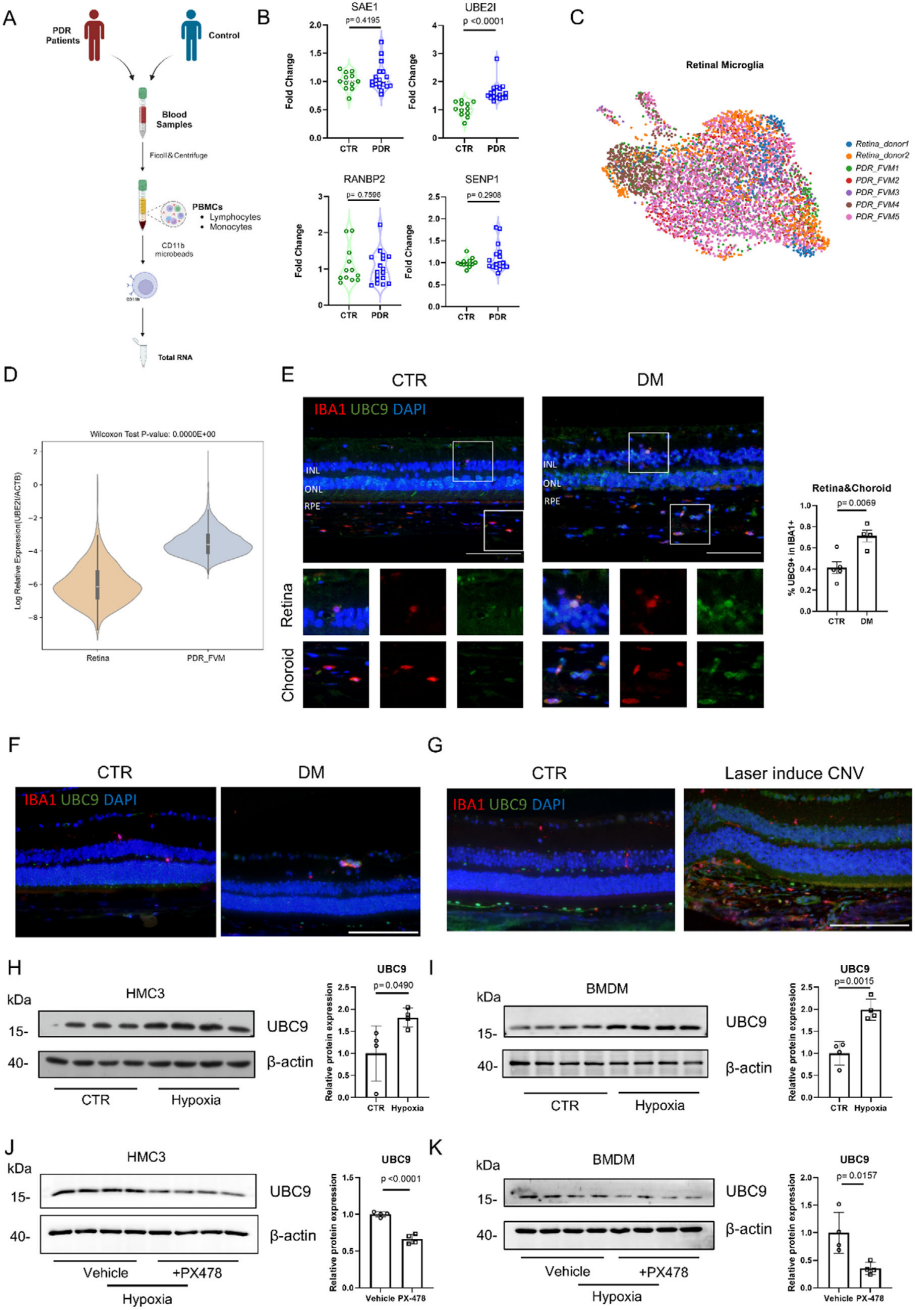
Elevated expression of UBC9 in MNPs is associated with pathological vascular disease in the retina. A) Schematic representation of sorted CD11b^+^ monocytes from human PBMCs. B) Expression levels of *SAE1*, *UBC9*, *RANBP2*, and *SENP1* in CD11b^+^ myeloid cells from control and proliferative diabetic retinopathy (PDR) patients (CTR, *n* = 12, PDR, *n* = 17). C) Integrated UMAP plot of retinal microglia from published data. Human adult retinas: 2 samples; Vitreous fibrovascular membranes (FVM) caused by proliferative diabetic retinopathy (PDR): 5 samples. D) Box plot showing the comparison of the expression levels of UBE2I between FVM and retinal microglia. E) Representative immunofluorescence of UBC9 co‐stained with IBA1 in the retina/choroid of control and diabetic patients (scale bar = 100 µm). The percentage of UBC9^+^ IBA1^+^cells were quantified (Control, *n* = 5; DM, *n* = 4). F) Representative images of UBC9^+^, IBA1^+^, and UBC9^+^ IBA1^+^cells in the retina/choroid of the control and diabetic mice (scale bar = 100 µm). G) Representative images of UBC9^+^, IBA1^+^, and UBC9^+^ IBA1^+^cells in the retina/choroid of control and laser‐induced CNV mice (scale bar = 100 µm). H, I) Quantification of UBC9 expression under hypoxic conditions in HMC3 cells and BMDMs by Western blot (*n* = 4). J, K) Quantification of UBC9 expression after PX478 treatment under hypoxic conditions in HMC3 cells and BMDMs by Western blot (*n* = 4). Unpaired two‐tailed Student's *t*‐test was used for statistical analysis (Wilcoxon test for scRNA‐seq analysis). Data are presented as mean ± SEM.

Pathological microvascular remodeling of the retina is partially ascribed to the dysfunction of MNPs.^[^ ^19^, ^20^ ^]^ To gain deeper insight into the possible involvement of protein SUMOylation in MNPs of retinal vasculopathy, blood samples were collected from control and proliferative diabetic retinopathy (PDR) patients. Following gradient density centrifugation, peripheral blood mononuclear cells (PBMCs) were isolated, and monocytes were purified by magnetic beads. *UBE2I* encodes the sole E2 SUMO‐conjugating enzyme UBC9. Strikingly, *UBE2I* mRNA level was substantially higher in circulating monocytes derived from PDR patients than from age‐ and sex‐matched controls, whereas the mRNA levels of other SUMOylation‐related genes such as *SAE1*, *RANBP2*, and *SENP1* remained unchanged (**Figure** **1**B). We then analyzed the expression of *UBE2I* in retinal microglial populations using publicly accessible single‐cell RNA sequencing datasets (EGAS00001004561, GSE165784). As expected, *UBE2I* expression was markedly upregulated in microglia from the PDR‐FVM (fibrovascular membrane) compared to normal donor retinas (Figure 1C,D). In consistent, immunofluorescence staining of posterior segment tissues from diabetic patients revealed elevated UBC9 in IBA1^+^ MNPs within the retina and choroid (Figure 1E). Similarly, UBC9 expression was increased in IBA1^+^ cells in the retinas of STZ‐induced diabetic mice (Figure 1F). We next established the laser‐induced choroidal neovascularization (CNV) model, where IBA1^+^ MNPs accumulated at CNV lesions, accompanying with elevated UBC9 expression (Figure 1G).

Hypoxia is a critical driver of retinal vasculopathy.^[^ ^21^ ^]^ To explore whether the hypoxic niche affects UBC9 expression, human microglia clone 3 (HMC3) cells and bone marrow‐derived macrophages (BMDMs) were cultured under normoxic or hypoxic (1% O₂) conditions. Hypoxic stimulation prominently increased UBC9 protein levels in both cell types (Figure 1H,I), while treatment with PX‐478, a specific inhibitor of HIF‐1α,^[^ ^22^ ^]^ markedly reduced hypoxia‐induced UBC9 expression in HMC3 and BMDM cells (Figure 1J,K). Moreover, other stimuli such as high‐glucose challenge, advanced glycation end products (AGE) treatment, and ER stress elicitation all similarly elevated UBC9 levels in BMDMs (Figure S1A–C, Supporting Information), supporting that upregulation of UBC9 in MNPs serves as a common feature of retinal vasculopathy.

### 2.2 Myeloid Specific Ablation of UBC9 Ameliorates Retinal Vasculopathy

**Figure 2:**
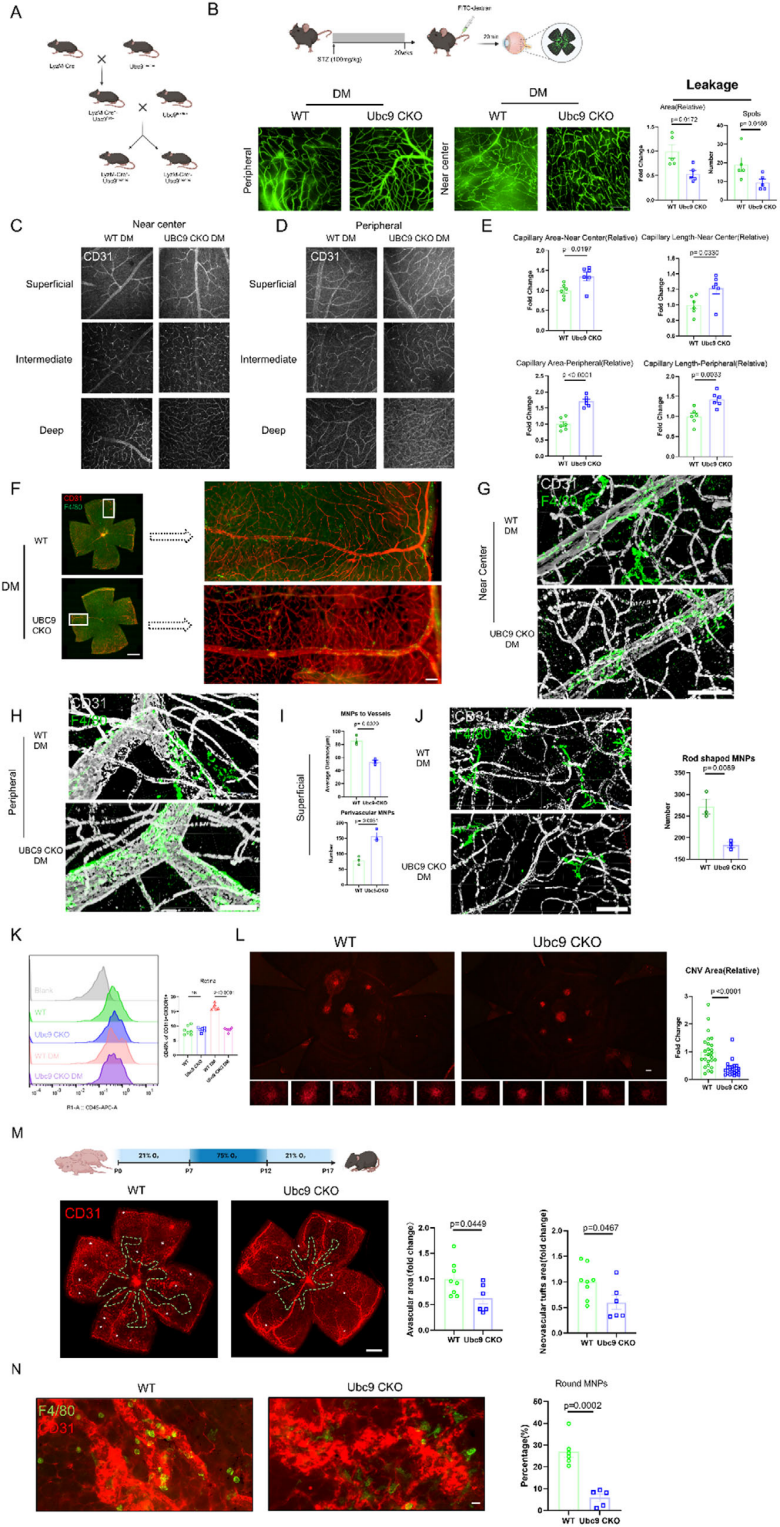
UBC9 contributes to the angioregulatory role of MNPs in the retina. A) Schematic illustration for the generation of Lyzm‐Cre^+^Ubc9^fl/fl^ mice by mating Lyzm‐Cre transgenic mice and Ubc9 flox mice. B) Reduced FITC‐dextran vascular leakage in the retina of Ubc9 CKO STZ‐DM mice (scale bar = 100 µm). FITC‐dextran leakage was quantified through the flat mount of the mouse retina by measuring the leaky area and spots (*n* = 5). C) Retina mounts of capillaries in three retinal layers in the near‐center region (scale bar = 100 µm). D) Retina mounts of capillaries in three retinal layers in the peripheral region (scale bar = 100 µm). E) Quantification of the total capillary density in all three layers of WT DM and Ubc9 CKO DM mice (*n* = 6). F) Whole scanning of the mouse retina stained with anti‐F4/80 and anti‐CD31 to visualize the association between MNPs and vasculature (scale bar = 1000 µm). G) 3D image of the spatial relationships between MNPs (F4/80^+^) and vasculature (CD31^+^) near the center between the WT DM group and Ubc9 CKO DM group (scale = 50 µm). H) 3D image of the spatial relationships of MNPs (F4/80^+^) and vasculature (CD31^+^) in the periphery of the WT DM group and Ubc9 CKO DM group (scale = 50 µm). I) Quantification of the average distance between the MNPs and the main vasculature in the superficial layer based on the whole scanning of the retina (*n* = 3). Perivascular MNPs were counted (*n* = 3). J) Representative image of rod‐shaped MNPs of a flat‐mount retina (scale = 50 µm). The number of rod‐shaped MNPs distributed in the superficial layer was counted in the entire scanning image of the retina (*n* = 3). K) The fluorescent intensity of CD45 in CD11b^+^ CXC3R1^+^ MNPs in the mouse retina was determined by flow cytometry (*n *= 6–7). L) Immunofluorescence of CD31 in the choroid mount of WT and Ubc9 CKO mice 1 week after laser coagulation, with quantification of the CNV area (scale = 100 µm) (*n* = 5). M) Whole scanning of the OIR mouse retina stained with anti‐CD31 to visualize the aberrant vascular patterns. The avascular area was delineated, and asterisks indicate neovascular tufts (scale bar = 1000 µm, WT *n *= 8, Ubc9 CKO *n* = 6). N) Neovascular tufts from P17 pup retina stained with anti‐F4/80 and anti‐CD31 (scale bar = 20 µm, WT *n* = 6, Ubc9 CKO *n *= 5). Unpaired two‐tailed Student's *t*‐test or one‐way ANOVA with Bonferroni's multiple comparisons test was used for statistical analysis. Data are presented as mean ± SEM.

To elucidate the role of MNPs‐expressed UBC9 in retinal microvascular diseases, myeloid‐specific Ubc9 knockout (Ubc9 CKO) mice were generated (**Figure** **2**A; Figure S2A, Supporting Information). We first established the diabetic retinopathy model in high‐dose streptozotocin induced diabetes mellitus (DM) mice. In the retina of 5‐month diabetic mice, FITC‐dextran leakage, indicative of vascular permeability, was found to be greatly reduced in the retina of Ubc9 CKO mice, which showed fewer leaky areas and spots compared to wild‐type (WT) littermate controls (Figure 2B).

Abnormal retinal vascularization associated with compromised visual function emerges as diabetic retinopathy progresses.^[^ ^23^ ^]^ Advanced stages of diabetic retinopathy are characterized by vessel loss, as evidenced by preclinical and clinical research.^[^ ^23^, ^24^ ^]^ Vessel density, a critical indicator of retinal blood supply, was significantly higher in the retinas of Ubc9 CKO mice after 5 months of diabetes, particularly in the intermediate and deep vascular layers in both central and peripheral regions (Figure 2C,D). The sum of superficial/intermediate/deep retinal capillaries was quantified by measuring the skeletonized capillary area and capillary length (Figure 2E). In age‐matched non‐diabetic controls, no significant differences in vessel density were observed (Figure S2B, Supporting Information).

We then sought to visualize the retinal MNPs using microscopic techniques. Interestingly, F4/80^+^ MNPs were less diffusely distributed within the superficial retinal layer of Ubc9 CKO diabetic mice (Figure 1F). 3D‐rendered confocal imaging of the diabetic retina revealed that superficial MNPs were in closer proximity to the main vasculature (vessel diameter ≥12 µm) (Figure 2G–I), and perivascular MNPs in direct contact with the main vessels were more abundant in the Ubc9 CKO group (Figure 2F–I). Furthermore, rod‐shaped MNPs, characterized by larger cell bodies and fewer intersections,^[^ ^25^ ^]^ were prominently decreased in the retinas of Ubc9 CKO diabetic mice (Figure 2J; Figure S2C,D, Supporting Information). By contrast, these distributional and morphological changes were not observed in MNPs within the intermediate and deep retinal layers (Figure S2E, Supporting Information).

Flow cytometry analysis was next performed to determine the activation status of MNPs. CD45^+^ CD11b^+^ CX3CR1^+^ cells, considered as inflammatory MNPs,^[^ ^21^ ^]^ were remarkably elevated in the WT diabetic retina, whereas they were lower in the Ubc9 CKO group (Figure 2K). Considering pathological ectopic neovascularization cannot be effectively mimicked by the STZ‐induced mouse model, we established the laser‐induced retinal injury model and examined the choroidal neovascular area to reflect the intensity of angiogenesis. Immunofluorescence analysis showed a marked reduction in CNV area in the Ubc9 CKO group compared to WT controls (Figure 2L), supporting the abrogated pro‐angiogenic capability of Ubc9 CKO MNPs. Ubc9 deficiency in MNPs inhibited vaso‐obliteration (manifested by avascular area) and the formation of neovascular tufts in the retina exposed to sequential oxygen challenge^[^ ^26^, ^27^ ^]^ (Figure 2M). Moreover, round‐shaped MNPs, representing macrophages activated by severe ischemia,^[^ ^28^, ^29^, ^30^ ^]^ were significantly reduced in the Ubc9 CKO retina of the oxygen‐induced retinopathy (OIR) model (Figure 2N)

Taken together, the above data indicated that UBC9 deficiency conferred retinal protective MNP phenotypes and hinted the possible MNP‐endothelial cell crosstalk.

### 2.3 UBC9 Bolsters the Pro‐Angiogenic Capability of MNPs

**Figure 3:**
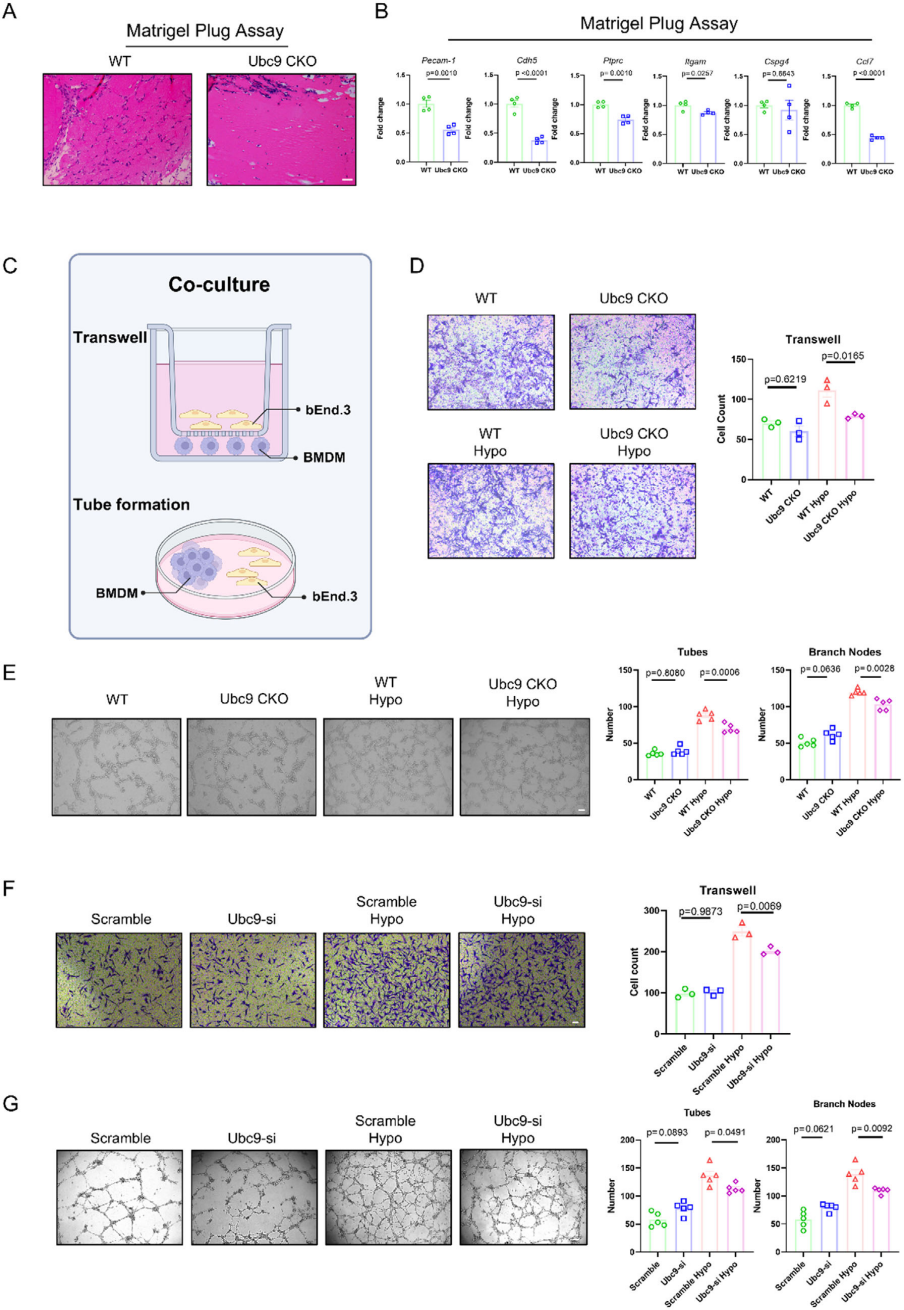
The pro‐angiogenic ability of MNPs is reduced upon Ubc9 depletion. A) Representative HE staining images of neovascularization in abdominal subcutaneous Matrigel plugs (scale bar = 50 µm). B) Quantification of neovascularization in the abdominal subcutaneous Matrigel plugs using RT‐qPCR analysis. Expression levels of *Pecam‐1*, *Cdh5*, *Ptprc*, *Itgam*, *Cspg4*, and *Ccl7* were evaluated in the plug (*n* = 4). C) Schematic representation of co‐culture experiment. D) Transwell assay images of bEnd.3 cells in co‐culture with BMDMs (scale = 20 µm). The migrated bEnd.3 cells were quantified and compared between WT and Ubc9 CKO groups (*n* = 3). E) Representative images of bEnd.3 cells and BMDMs co‐seeded in Matrigel (scale = 20 µm). The number of tubes and branched points formed were counted for statistical analysis (*n* = 5). F) Transwell assay images of HRMECs co‐cultured with HMC3 cells (scale = 20 µm). Migrated HRMECs were evaluated between the scramble and Ubc9 siRNA groups (*n* = 3). G) Representative images of HRMECs and HMC3 cells co‐seeded in Matrigel (scale = 20 µm). The number of tubes and branched points formed was counted to evaluate tube/cord formation ability (*n* = 5). Unpaired two‐tailed Student's *t*‐test or one‐way ANOVA with Bonferroni's multiple comparisons test was used for statistical analysis. Data are presented as mean ± SEM.

Aiming to further test the pro‐angiogenic capability of WT versus UBC9 deficient MNPs, the in vivo subcutaneous Matrigel plug assay was performed.^[^ ^31^ ^]^ Angiogenesis was assessed by HE staining and RT‐qPCR analysis of extracted Matrigel plugs. In line with the laser‐injury and OIR models, the Ubc9 CKO group showed reduced vascular density and markers of neovascular formation (*Pecam‐1*, *Cdh5*, *Ptprc*, *Itgam*, *Cspg4*, *Ccl7, Vegfa, and Adgre1*), when compared to WT group (**Figure** **3**A,B; Figure S3A, Supporting Information). Moreover, we co‐cultured bEnd.3 endothelial cells with BMDMs in either a Transwell chamber or Matrigel‐coated plate (Figure 3C). Under hypoxic conditions, WT BMDMs promoted endothelial cell invasion (Figure 3D) and tube/cord formation (Figure 3E), whereas these effects were mitigated in the Ubc9 CKO group (Figure 3D,E). Similarly, endothelial cells co‐cultured with Ubc9 conditional knockout BMDMs exhibited reduced migratory capacity after lipopolysaccharide (LPS) or IL‐4 pretreatment (Figure S3B, Supporting Information) under hypoxic conditions, suggesting that the regulatory effect of UBC9 on the pro‐angiogenic capability of macrophages is irrelevant to the M1/M2 phenotypes. In addition, VEGFA supplementation failed to eliminate the disparities in pro‐angiogenic capacities of WT and Ubc9 CKO BMDMs (Figure S3E,F, Supporting Information), while Ranibizumab (anti‐VEGF antibody) treatment reduced macrophage pro‐angiogenic potential, with more prominent efficacy being observed in Ubc9 CKO group (Figure S3G,H, Supporting Information).

We then validated the efficacy of UBC9 siRNA in HMC3 microglial cells and subsequently co‐cultured them with human retinal microvascular endothelial cells (HRMECs) (Figure S3C,D, Supporting Information). Consistent with the above findings, HRMECs co‐cultured with Ubc9‐knockdown HMC3 displayed diminished migratory abilities and tube/cord formation under hypoxic conditions (Figure 3F,G). Collectively, these results demonstrated that UBC9 ablation in MNPs impairs their pro‐angiogenic capacity.

### 2.4 UBC9 Deficiency Restrains VEGFA Secretion from Hypoxic Macrophages

**Figure 4:**
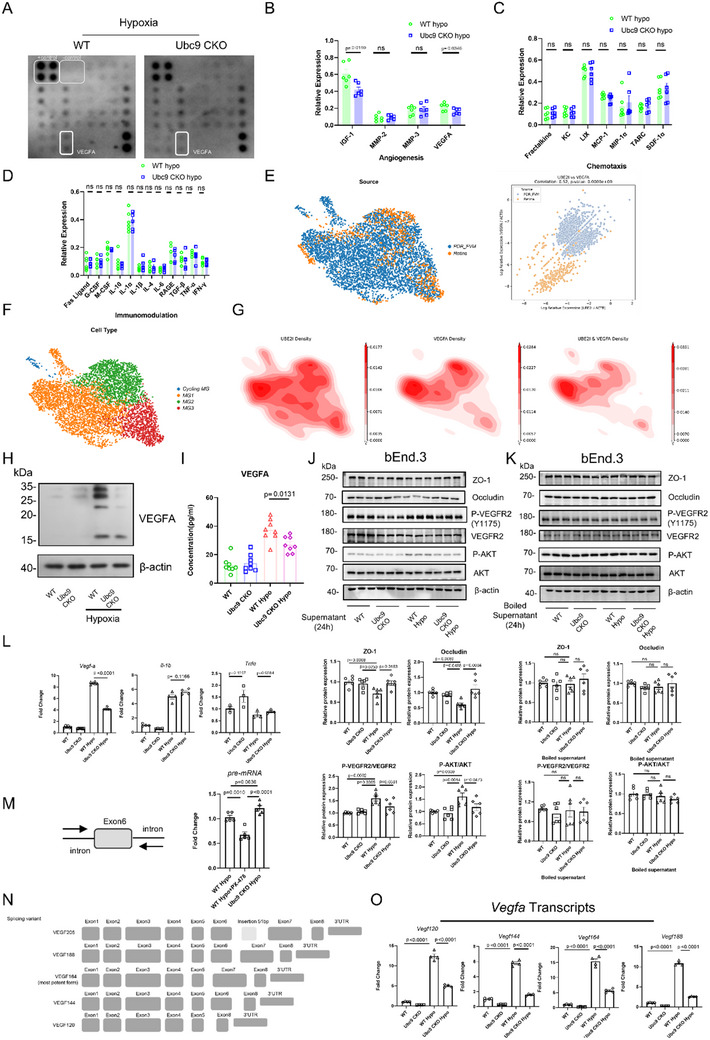
Depletion of UBC9 reverses the phenotype of pro‐angiogenic MNPs with lower VEGFA levels under hypoxic conditions. A) Representative images of the cytokine arrays of WT and Ubc9 CKO BMDMs under hypoxic conditions. B–D) Relative expression of the indicated cytokines (Fas ligand, Fractalkine, G‐CSF, IFN‐γ, IGF‐1, IL‐10, IL‐1α, IL‐1β, IL‐4, IL‐6, KC, LIX, MCP‐1, M‐CSF, MIP‐1α, MMP‐2, MMP‐3, RAGE, TARC, SDF‐1 α, TGF‐β, TNF‐α, and VEGFA) (*n* = 6). E) Integrated UMAP plot of retinal microglia from healthy donors and FVM‐PDR patients; Correlation between UBE2I and VEGFA expression levels in the retinal microglia. F) Integrated UMAP plot of heterogenous retinal microglia subpopulations. G) Weighted density plot of UBE2I and VEGFA in the retinal microglia subpopulations. H) Expression of VEGFA in the WT and Ubc9 CKO BMDMs under normoxic and hypoxic conditions. I) ELISA was performed to measure VEGFA levels in the culture medium of BMDMs from WT and Ubc9 CKO mice (*n* = 8). J–K) Endothelial cell junction proteins (ZO‐1, Occludin), p‐VEGFR2, and p‐AKT were measured by Western blot in bEnd.3 cells after 24 h treatment with the indicated BMDM supernatants (J) or boiled supernatants (K) (*n* = 6). L) Expression levels of *Vegfa* (*n* = 6), *Il‐1b* (*n* = 4), and *Tnfα* (*n* = 3) in WT and Ubc9 CKO groups. M) Expression levels of *Vegfa* pre‐mRNA detected by primers flanking introns near exon 6. The transcription of pre‐mRNA in WT+PX‐478 hypoxic and Ubc9 CKO hypoxic BMDMs were compared to the WT hypoxic group (*n* = 5). N) Schematic illustration of m*Vegfa* major transcripts. O) Expression levels of 4 different transcript variants of *Vegfa* (*Vegf120*, *Vegf144*, *Vegf164*, and *Vegf188*) in WT and Ubc9 CKO groups (*n* = 4). Unpaired two‐tailed Student's *t*‐test or one‐way ANOVA with Bonferroni's multiple comparisons test was used for statistical analysis. Data are presented as mean ± SEM.

Given both Transwell and Matrigel co‐culture assays unveiled phenotypic changes of endothelial cells, we deduced that UBC9 deficient MNPs may exhibit distinct secretome. BMDMs were subjected to hypoxic stimulation and harvested to perform a “cytokine array” to identify differential secretory mediators between the WT and Ubc9 CKO groups (**Figure** **4**A). Notably, VEGFA and IGF1 levels were significantly reduced in Ubc9 CKO BMDMs (Figure 4B), while levels of various chemokines and inflammatory cytokines, including FasL, Fractalkine, MCP‐1, MIP‐1α, G‐CSF, M‐CSF, IFN‐γ, IL‐1α, IL‐1β, IL‐4, IL‐6, IL‐10, and TNF‐α, remained unaltered (Figure 4C,D). Correlation analysis revealed a positive co‐expression pattern between *UBE2I* and *VEGFA* in retinal microglia (Figure 4E). By classifying microglia into four clusters (Figure 4F), it was found that *VEGFA* and *UBE2I* are mainly co‐expressed in MG1 and MG2 subpopulations, which represent the activated microglial state (Figure 4G; Figure S4A,B, Supporting Information).

Furthermore, Western blot results confirmed the elevation of VEGFA in BMDMs upon hypoxic stimulation, whereas UBC9 deficient BMDMs produced much less VEGFA (Figure 4H). Using ELISA assay, we demonstrated that UBC9 deficiency decreased VEGFA content within the supernatant of hypoxic BMDMs (Figure 4I). Next, the effects of conditioned BMDM culture medium on endothelial cells were evaluated by supernatant transfer. Supernatant from WT BMDMs reduced the expression of tight junction protein (ZO‐1, Occludin), and promoted VEGFR2 and AKT phosphorylation in bEnd.3 endothelial cells (Figure 4J). Conversely, supernatants from hypoxic Ubc9 CKO BMDMs attenuated vascular permeability by preserving ZO‐1 and Occludin levels and suppressing VEGFR2 and AKT phosphorylation (Figure 4J). Of note, the abovementioned effects were diminished when boiled supernatant was used, ruling out the contribution of heat‐stable metabolites (Figure 4K).

Considering Western blot results indicated multiple bands of VEGFA, we decided to check it at the mRNA level. Consistent with cytokine array results, RT‐qPCR analysis corroborated the reduction of total mature *Vegfa* mRNA levels in Ubc9 CKO BMDMs (Figure 4L; Figure S4C, Supporting Information). The expression of inflammation‐associated genes such as *Il‐1b* and *Tnfα* remained unchanged (Figure 4L). Concurrently, Ubc9 deficiency downregulated the transcription levels of *Angptl4*, *Pdgfa*, *Pdgfb*, and *Mmp9*, whereas exerting no perceptible impact on *Slit2*, *Fgf1*, *Fgf2*, and *APLN* transcription when compared with the WT group (Figure S4D, Supporting Information). Treatment with HIF‐1α inhibitor PX478 decreased *Vegfa* pre‐mRNA levels, interestingly, *Vegfa* pre‐mRNA levels were comparable between hypoxic Ubc9 CKO and WT BMDMs, suggesting an alternative HIF‐1α independent mechanism of VEGFA regulation mediated by UBC9 (Figure 4M). *Vegfa* pre‐mRNA engenders several splicing isoforms that can be discriminated by tailored primer pairs (Figure 4N).^[^ ^32^ ^]^ Analysis of *Vegfa* transcript variants revealed hypoxia‐induced upregulation of *Vegf120*, *Vegf144*, *Vegf164*, and *Vegf188* mRNAs in BMDMs, which were all downregulated by UBC9 deficiency (Figure 4O). Piecing these lines of evidence together, our data pinpointed to a post‐transcriptional boosting effect of UBC9 on VEGFA production.

### 2.5 UBC9 Facilitates the SUMOylation of FUS

**Figure 5:**
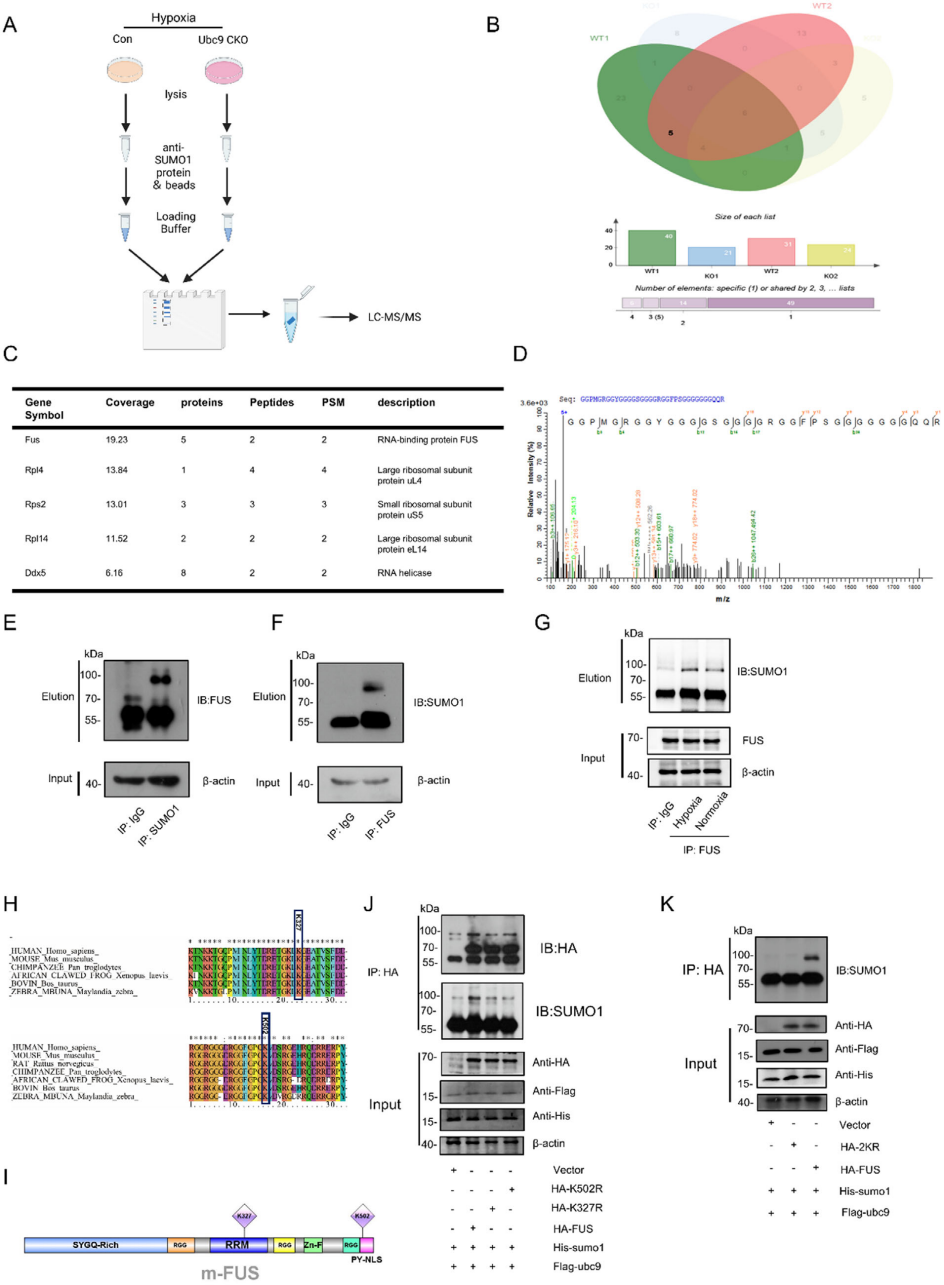
UBC9 mediates SUMOylation of FUS in MNPs. A) Schematic illustration of the experimental design for mass spectrometry after immunoprecipitation with the anti‐SUMO1 antibody of WT and Ubc9 CKO BMDM lysates. B) Venn diagram illustrating the intersection results obtained from the WT and Ubc9 CKO groups from the parallel experiments. C) Five proteins identified by IP‐MS. D) Mass spectrogram showing peptides identified from FUS. E) Western blot of the Co‐IP lysates showing the interaction between SUMO1 and FUS, using anti‐SUMO1. F) Western blot results for Co‐IP between FUS and SUMO1, using anti‐FUS. G) The interaction between SUMO1 and FUS in BMDMs under normoxic and hypoxic conditions was detected by Co‐IP assays. H) Sequence conservation analysis through multiple sequence alignment of amino acid residues of FUS across different species on lysine327 (K327) and lysine502 (K502). I) Schematic diagram of mouse FUS domains and motifs. J) Indicated plasmid‐transfected HEK‐293T cells were subjected to Co‐IP and Western blot to detect SUMOylated FUS after K327R or K502R point mutation. K) Interaction of K327R/K502R double‐mutant FUS with SUMO1 was detected by Co‐IP and Western blot in adenovirus‐transduced HEK‐293T cells.

UBC9 serves as the major E2 conjugating enzyme that mediates substrate SUMOylation.^[^ ^11^ ^]^ To obtain a deeper mechanistic insight, we performed protein co‐immunoprecipitation followed by mass spectrometry to identify SUMO1‐interacting proteins in hypoxic BMDMs (**Figure** **5**A). By comparing the differentially expressed proteins between WT and Ubc9 CKO groups, we narrowed down to five candidate SUMOylation substrates (Figure 5B). FUS, the RNA‐binding protein (RBP) involved in multiple processes of RNA metabolism, was selected as the potential SUMOylation target (Figure 5C,D).

Cross co‐immunoprecipitation experiments with either anti‐SUMO1 or anti‐FUS antibodies validated the band shift caused by SUMO1‐attached FUS (Figure 5E,F). Additionally, SUMOylation of FUS protein was enhanced in BMDM under hypoxic stimulation (Figure 5G). Using bioinformatics tools, including SUMOplot and GPS‐SUMO, we predicted lysine327 (K327) and lysine502 (K502) as candidate SUMOylation sites on FUS (Figure S5A,B, Supporting Information). Sequence analysis found that these residues are highly conserved across species (Figure 5H). While K327 is located within the RNA recognition motif (RRM), K502 resides between the nuclear translocation motif (PY‐NLS) and an arginine‐glycine‐rich (RGG) domain (Figure 5I). Mutagenesis and Ubc9‐expressing vector co‐transfection studies revealed that either K327R or K502R mutation partially abated FUS SUMOylation (Figure 5J). Subsequently, a double mutant FUS (2KR) construct was generated, and transfection of FUS‐2KR completely abolished the SUMOylation of FUS (Figure 5K). Therefore, we identified FUS as the downstream substrate of UBC9‐mediated protein SUMOylation, occurring at K327 and K502 sites.

### 2.6 SUMOylation Defect Confers Enhanced Capacity of FUS to Destabilize VEGFA Transcripts

**Figure 6:**
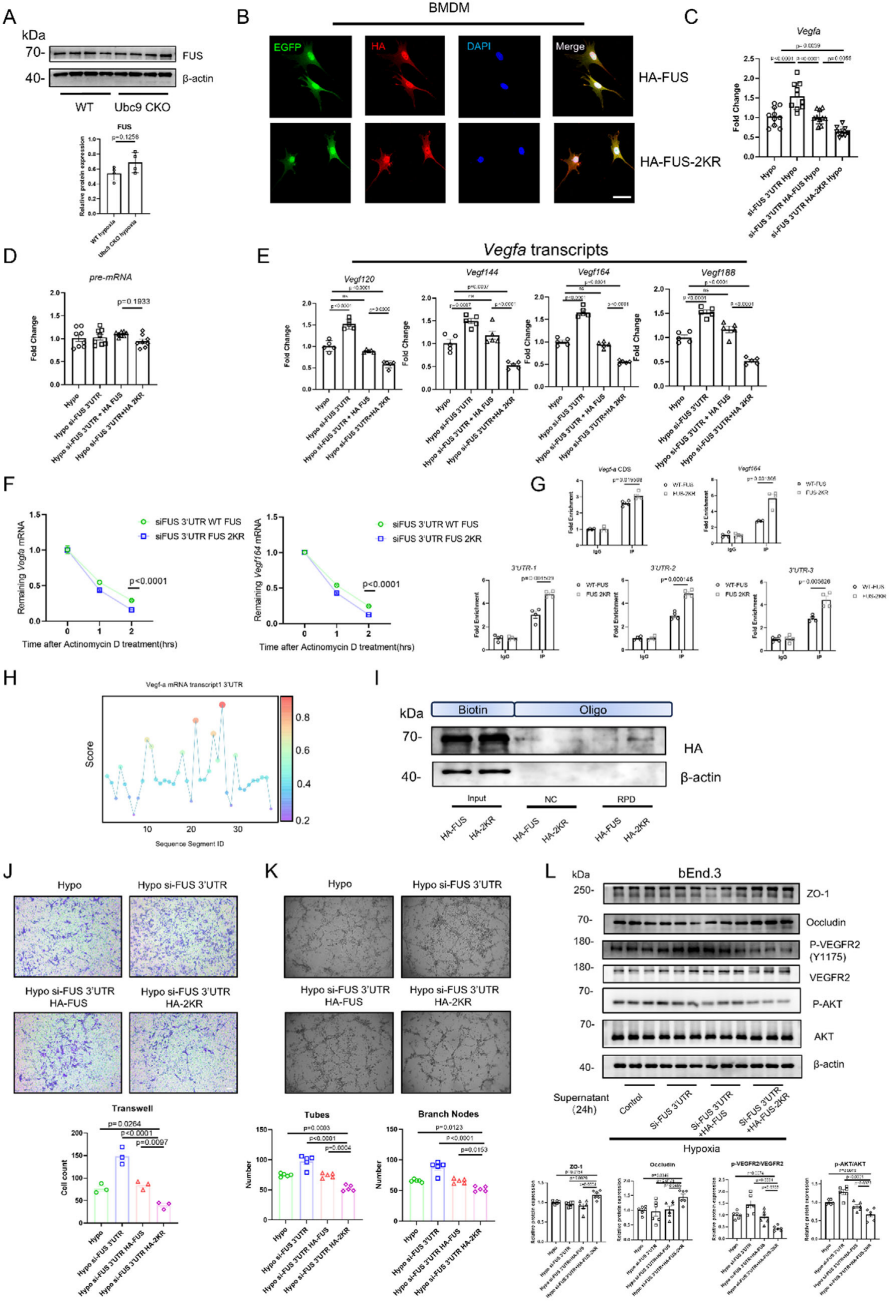
DeSUMOylation of FUS mitigates the pro‐angiogenic activity of MNPs. A) Quantification of FUS expression under hypoxic conditions in WT and Ubc9 CKO BMDMs by Western blot (*n* = 4). B) Representative images of nuclear localization of FUS‐WT and FUS‐2KR in adenovirus‐transduced BMDMs (scale bar = 20 µm). C) *Vegfa* expression levels were determined by RT‐qPCR in the indicated treatment groups (*n* = 10). D,E) Expression levels of *Vegfa* pre‐mRNA (D, *n* = 8) and 4 splicing variants (*Vegf120*, *Vegf144*, *Vegf164*, and *Vegf188*) (E, *n *= 5) were quantified using RT‐qPCR in the indicated treatment groups. F) mRNA stability of *Vegfa* and *Vegf164* was measured in FUS‐WT and FUS‐2KR BMDMs after treatment with 10 µg mL^−1^ Actinomycin D (*n *= 5–6). G) RNA immunoprecipitation of FUS‐WT and FUS‐2KR in BMDMs. The products were subjected to RT‐qPCR using the indicated primers, and the efficiency was evaluated by fold‐enrichment (*n* = 4). H) Prediction of binding of FUS protein and *Vegfa* 3′UTR based on the RBPsuite. I) RNA pull‐down was performed with a biotin‐labeled probe flanking the 3′UTR of *Vegfa* mRNA to assess the binding of FUS‐WT and FUS‐2KR to *Vegfa* mRNA. J) Transwell assays were performed to evaluate the migration of bEnd.3 cells toward adenovirus‐transduced BMDMs in the lower chamber under hypoxic conditions (scale = 20 µm, *n* = 3). K) Tube formation assays were performed to detect the angiogenic potential of bEnd.3 cells by co‐seeding with the indicated adenovirus‐transduced BMDMs in Matrigel under hypoxic conditions (scale = 20 µm, *n* = 5). L) Western blot was performed to measure the protein levels of ZO‐1, Occludin, p‐VEGFR2, and p‐AKT in bEnd.3 cells after treatment with the indicated supernatant of BMDMs (*n* = 6). Unpaired two‐tailed Student's *t*‐test or one‐way ANOVA with Bonferroni's multiple comparisons test was used for statistical analysis. Data are presented as mean ± SEM.

SUMOylation fine‐tunes protein function through various aspects. Of note, the expression of FUS remained unaltered between WT and Ubc9 CKO BMDMs (**Figure** **6**A). Considering the proximity of the K502 site to the N‐terminal NLS motif (Figure 5I), we investigated the subcellular distribution of FUS in BMDMs. However, both Ubc9 conditional knockout and FUS mutation (K327R and K502R) did not affect the nuclear translocation of FUS in BMDMs (Figure 6B; Figure S5D, Supporting Information).

Following the depletion of endogenous FUS with 3′UTR targeted siRNA, BMDM cells were transduced with either wild‐type (FUS‐WT) or mutant (FUS‐2KR) FUS‐encoding viruses (Figure S6A,B, Supporting Information). Strikingly, mature *Vegfa* mRNA level was increased by FUS knockdown and lower in FUS‐2KR transduced BMDMs compared to FUS‐WT counterparts (Figure 6C). In contrast, transcription levels of *Tnfα* and *Il‐1b* remained unchanged (Figure S6C, Supporting Information). *Vegfa* pre‐mRNA levels were also not affected by FUS knockdown or SUMOylation defect (Figure 6D), while *Vegfa* splicing variants (*Vegf120/144/164/188*) were downregulated in the FUS‐2KR group (Figure 6E).

*Vegf164* is the dominant and active form of *Vegfa* transcripts, making it a primary focus of subsequent investigations. *Vegfa/Vegf164* mRNA stability was measured in hypoxic BMDMs treated with the transcription inhibitor Actinomycin D. *Vegfa/Vegfa164* mRNA exhibited reduced stability in Ubc9 CKO BMDMs (Figure S6D, Supporting Information) and in cells transduced by FUS‐2KR (Figure 6F), while *Vegfa/Vegfa164* mRNA stability increased upon FUS knockdown (Figure S6E, Supporting Information). RNA immunoprecipitation (RIP) experiments demonstrated that FUS‐2KR exhibited higher binding affinity to *Vegfa*/*Vegfa164* mRNA, especially the 3′ untranslated region (3′UTR) (Figure 6G). Using RBPsuite,^[^ ^33^ ^]^ we predicted 3′UTR AU‐rich region as a conserved FUS binding site (Figure 6H; Figure S6F,G, Supporting Information). A pull‐down assay with biotin‐labeled probes confirmed that FUS binds to the *Vegfa* 3′UTR AU‐rich region, with stronger binding affinity observed for FUS‐2KR (Figure 6I).

To explore the functional relevance of FUS and SUMOylated FUS in MNP‐induced angiogenesis, bEnd.3 endothelial cells were co‐cultured with FUS deficient, FUS‐WT and FUS‐2KR BMDMs. FUS mutations (K327R and K502R) in BMDMs significantly attenuated endothelial migration and tube/cord formation in co‐culture assays (Figure 6J,K). Additionally, compared to the FUS‐WT group, supernatant from FUS‐2KR BMDMs resulted in higher expression of cell junction proteins (ZO‐1, Occludin) and diminished VEGFR2 pathway (Figure 6L).

Together, these results established that deSUMOylation of FUS impedes the pro‐angiogenic function of MNPs by promoting the instability of *Vegfa* mRNA transcripts.

### 2.7 UBC9 Inhibition Synergizes with Anti‐VEGF Therapy

**Figure 7:**
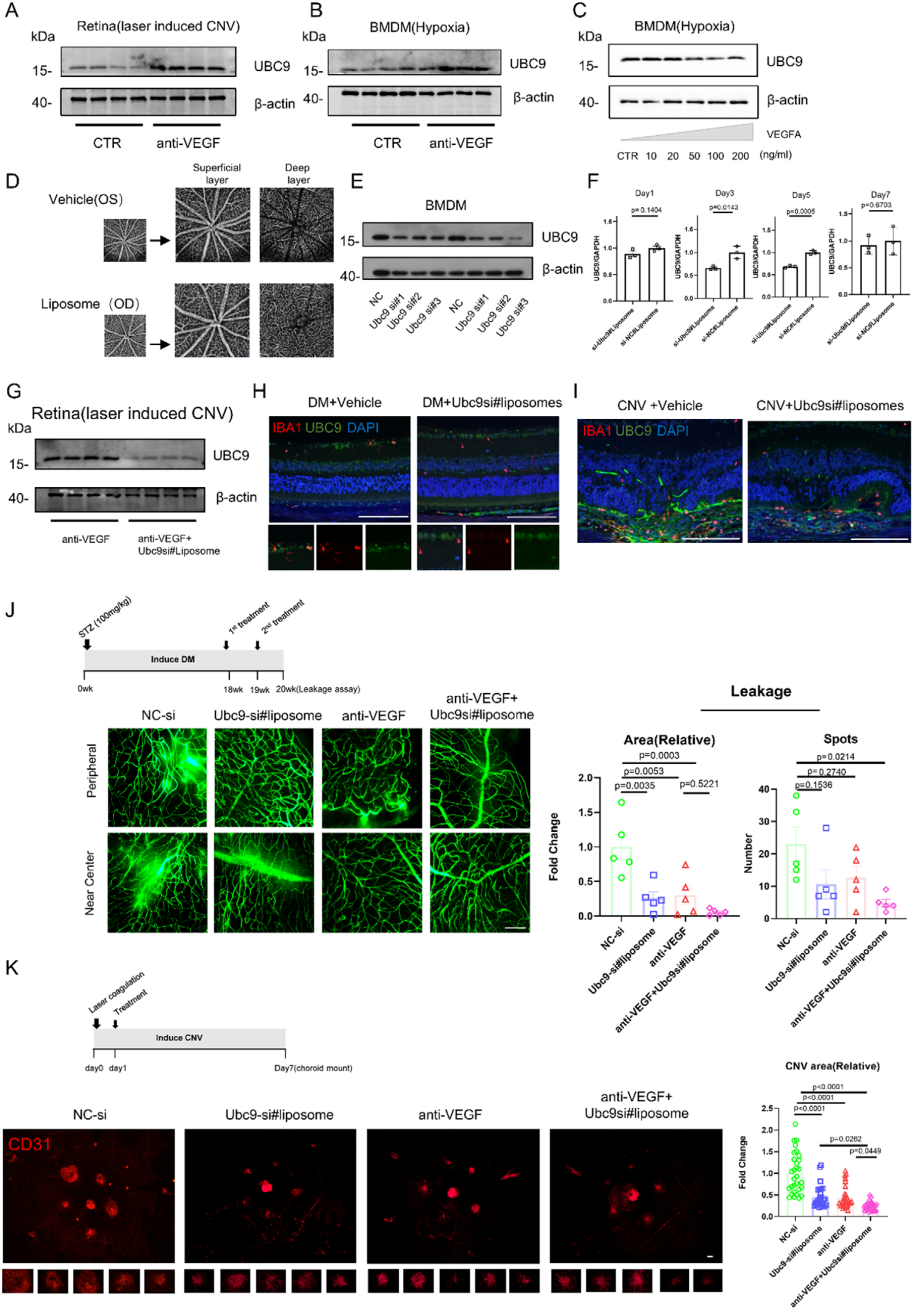
Abating UBC9 reinforces the effects of anti‐VEGF treatment. A) Retinal protein levels of UBC9 were quantified and compared between the CNV+anti‐VEGF treatment group and the CNV control group (*n *= 4). B) Quantification of UBC9 protein levels of the control and anti‐VEGF group in hypoxic BMDMs by Western blot (*n* = 4). C) Quantification of UBC9 protein levels of the control and VEGFA treatment group in BMDMs by Western blot (*n* = 4). D) Optical coherence tomography angiography images of mouse retina after PBS buffer or liposome (prepared in PBS) intravitreal injection. E) The knockdown efficiency of Ubc9 siRNA was evaluated in BMDMs by Western blot. F) The knockdown efficiency of Ubc9 siRNA#liposome at day1/day3/day5/day7 after intravitreal injection was evaluated in the mouse retina by Western blot (*n* = 3). G) The knockdown efficiency of Ubc9 siRNA#liposome in the CNV retina after the intravitreal injection of anti‐VEGF (*n* = 4). H) Representative images of UBC9 and IBA1 immunofluorescence in the retina/choroid of diabetic mice and diabetic mice treated with Ubc9 siRNA#liposome. I) Representative images of UBC9 and IBA1 immunofluorescence in the retina/choroid of mice with CNV and CNV mice with Ubc9 siRNA#liposome treatment. J) Vascular permeability was measured using a flat mount of diabetic mouse retina in the indicated treatment groups (Control, Ubc9si#liposome, anti‐VEGF, and a combination of Ubc9si#liposome and anti‐VEGF) (scale bar = 100 µm). The relative areas and spots of FITC‐dextran leakage were quantified (*n* = 5). K) Representative immunofluorescence images of CD31 in the choroid mount of the indicated treatment groups (Control, Ubc9si#liposome, anti‐VEGF, and a combination of Ubc9si#liposome and anti‐VEGF) (scale bar = 100 µm). The choroidal neovascularization area was quantified (*n* = 6). Unpaired two‐tailed Student's *t*‐test or one‐way ANOVA with Bonferroni's multiple comparisons test was used for statistical analysis. Data are presented as mean ± SEM.

Ranibizumab is a commonly prescribed drug targeting angiogenesis in ocular fundus diseases.^[^ ^34^ ^]^ Yet, we found that ranibizumab treatment elevated retinal UBC9 expression following laser photocoagulation (**Figure** **7**A). Similarly, UBC9 protein levels increased in hypoxic BMDMs upon ranibizumab treatment (Figure 7B). A higher concentration of recombinant VEGFA reduced UBC9 expression, whereas VEGFR2 inhibition by SU5408 abolished such effect (Figure 7C; Figure S7, Supporting Information). Altogether, these findings hint that anti‐VEGF therapy may inadvertently contribute to a self‐reinforcing cycle involving UBC9 overexpression.

To explore the possibility of a combinatorial strategy with Ubc9 inhibition and anti‐VEGF therapy, we first tested the knockdown efficiency of Ubc9 siRNA (Figure 7D) and prepared the siRNA#3 loaded liposomes (siRNA#liposome) in vitro (Figure S8A–D, Supporting Information). Intravitreal injection of siRNA#liposome proved safe to retinal vascular architecture (Figure 7E; Figure S8F,G, Supporting Information), and UBC9 expression was decreased on day 3 and day 5 following siRNA#liposome treatment but recovered by day 7 (Figure 7F; Figure S8E, Supporting Information). The Ubc9 siRNA#liposome also effectively reduced UBC9 expression in the CNV retina after administration of ranibizumab (Figure 7G). Meanwhile, the Ubc9 siRNA#liposome could suppress UBC9 expression in retinal MNPs under both diabetic and CNV conditions (Figure 7H,I).

At last, the therapeutic efficacy of combined Ubc9 siRNA#liposomes and ranibizumab was compared with monotherapies. All treatments substantially alleviated retinal vascular leakage in STZ‐induced diabetic mice, with the combination treatment showing optimal efficacy (Figure 7J). In consistent, the combination therapy provided superior suppression of CNV formation in laser‐induced mice compared to either of the monotherapy (Figure 7K).

Collectively, these results endorsed the applicability of Ubc9 inhibition in retinal vasculopathies. When combined with anti‐VEGF treatment, it may reduce the required anti‐VEGF dosage, potentially mitigating adverse effects and improving clinical outcomes.

## 3 Discussion

**Figure 8:**
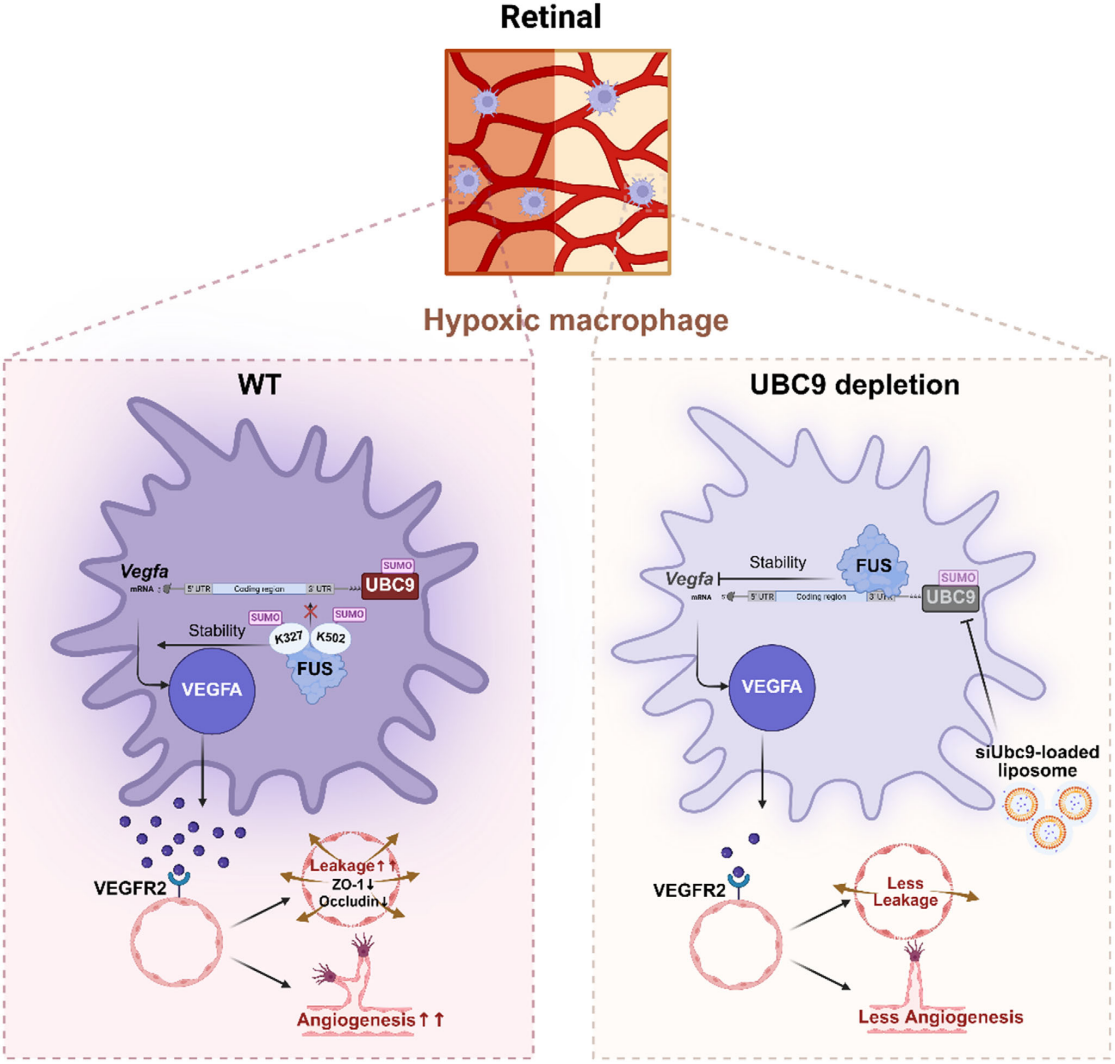
Schematic illustration of the pro‐angiogenic effect of UBC9 in hypoxic macrophages. In hypoxic macrophages, elevated expression of UBC9 facilitates the SUMOylation of FUS (on lysine residue of the RNA binding motif), thereby directly inhibiting the degradation of *Vegfa* mRNA and conferring the pro‐angiogenic capacity. In parallel, anti‐VEGF therapy upregulates UBC9 in a compensatory manner, rendering Ubc9 inhibition a potential treatment approach for retinal vasculopathy.

In this study, we illuminated the pivotal role of UBC9‐mediated SUMOylation in modulating the pro‐angiogenic capacity of MNPs in retinal vascular diseases. SUMOylation of FUS at lysine residues 327 and 502 disturbs its interaction with the 3′UTR of *Vegfa* mRNA, thereby stabilizing *Vegfa* transcripts. Importantly, the combined therapeutic strategy of UBC9 inhibition and anti‐VEGF treatment offers a promising approach for effectively treating retinal vasculopathy (Figure 8).

MNPs, including circulating/infiltrating and resident subpopulations, are intimately engaged with vascular remodeling.^[^ ^35^ ^]^ Microglia gets close contact with retinal vessels.^[^ ^8^, ^36^ ^]^ Mixed polarized microglia, including inflammatory M1 and anti‐inflammatory M2 subtypes, contribute to retinal neovascularization and retinal degeneration,^[^ ^10^ ^]^ the concept of which was also confirmed by our results (Figures S3B and S4C, Supporting Information). Intriguingly, hypoxia affects macrophages in a specific manner. Oxygen deprivation promotes inflammatory M1 phenotype, whereas lactate generated from anaerobic metabolism favors the M2 program.^[^ ^37^ ^]^ During placental angiogenesis and tumor growth, hypoxia induces VEGFA secretion in alternatively activated macrophages.^[^ ^38^, ^39^ ^]^ In advanced diabetic retinopathy, lower oxygen tension is commonly detected, signifying a hypoxic retinal microenvironment.^[^ ^40^ ^]^ Hypoxia enables the production of IL‐1β/IL‐6/VEGFA from myeloid cells, and HIF‐1α knockout ameliorates pathological angiogenesis in a mouse model of oxygen‐induced retinopathy.^[^ ^41^ ^]^ Nevertheless, an initial reduction in vessel density is observed as early as 4 weeks following STZ injection, predisposing the retina to a progressive ischemic microenvironment.^[^ ^24^ ^]^ Meanwhile, low glucose levels could also promote the accumulation of HIF‐1α in the absence of hypoxia, subsequently triggering the secretion of angiogenic factors.^[^ ^42^ ^]^ In our study, UBC9 could be upregulated by hypoxic stimuli, glucose challenge, AGE treatment, or ER stress elicitation (Figure 1H,I; Figure S1A–C, Supporting Information). UBC9 deficient hypoxic MNPs manifested reduced production of VEGFA rather than inflammatory mediators (Figure 4B,K). Besides, unlike the transcriptional regulation of HIF‐1α, UBC9, and FUS mainly affect the mRNA stability of *Vegfa*.

Previous studies have shown that UBC9 plays a part in macrophage polarization.^[^ ^13^, ^43^ ^]^ Our earlier work demonstrated the essential function of UBC9 in facilitating IL‐4‐induced M2 polarization of macrophages.^[^ ^13^ ^]^ In prostate cancer, UBC9 deficiency has been linked to enhanced macrophage activation and antigen presentation.^[^ ^44^ ^]^ Here, we observed that UBC9 deficiency decreases angiogenic factor VEGFA in hypoxic macrophages, adding a new dimension of macrophage plasticity. The downregulation of VEGFA may interrupt the autocrine VEGFA/VEGFR1 loop,^[^ ^21^ ^]^ explaining the dispersed rod‐shaped macrophages observed in the diabetic mouse retina and the compensatory upregulation of UBC9 upon anti‐VEGF treatment. Given the proximity of microglia/macrophages to vascular endothelial cells, the phenotypic changes induced by UBC9 deficiency contributed to improved vascular integrity and reduced pathological neovascularization. Collectively, these findings demonstrated that UBC9 regulates the pro‐angiogenic capacity while exerting minimal influence on inflammatory response in hypoxic macrophages.

FUS is a DNA/RNA‐binding protein implicated in the progression of amyotrophic lateral sclerosis and frontotemporal dementia (ALS/FTD). Mutations in FUS facilitate phase separation, leading to the accumulation of stress granules and other ribonucleoproteins. These mutations predominantly cluster within the proline‐tyrosine nuclear localization signal (PY‐NLS) at the C‐terminus and alter the nuclear import of FUS proteins.^[^ ^45^, ^46^ ^]^ Previous studies have shown that circular RNA CircFndc3b promotes the degradation of FUS protein, which subsequently increases the expression of HIF1α and its downstream target VEGFA.^[^ ^47^, ^48^ ^]^ In consistent, our findings revealed that silencing FUS markedly elevated *Vegfa* mRNA levels in macrophages (Figure 6C,E). In this study, we uncovered that FUS undergoes SUMOylation at two critical lysine residues, K327 and K502. K327 is located within the RRM motif, while K502 resides near the RGG region. These domains participate in FUS's bindings with mRNA.^[^ ^49^ ^]^ Indeed, the nuclear import of FUS is not affected while the RNA binding affinity is suppressed by SUMOylation. DeSUMOylated FUS exhibited enhanced interaction with the 3′UTR of *Vegfa* mRNA, thereby destabilizing *Vegfa* transcripts under hypoxic conditions. Therefore, SUMOylation constitutes an additional mechanistic layer to control the activity of FUS.

Anti‐VEGF therapy is the prevalent treatment for retinal vascular diseases. However, clinical practice highlights the unmet need to reduce anti‐VEGF dosage and enhance therapeutic outcomes.^[^ ^34^ ^]^ In one respect, incomplete response in nAMD patients reveals a dilemma for anti‐VEGF‐neutralizing antibodies.^[^ ^50^ ^]^ Persistent disease activity may be attributed to disease heterogeneity, genetic polymorphisms, compensatory upregulation of other pro‐angiogenic factors, and suboptimal treatment dosing associated with PRN (pro re nata) protocol. Additionally, long‐term plus high‐potency utilization of these neutralizing antibodies raises ophthalmological and systemic risks.^[^ ^51^ ^]^ Continued efforts have been made to improve the therapeutic efficacy through combination therapy. Corticosteroids are commonly co‐administered in diabetic macular oedema, though extended treatment cycles often exacerbate adverse effects.^[^ ^52^, ^53^ ^]^ OPT‐302 (VEGFC/D inhibitor) in combination with ranibizumab achieves primary outcomes with superior BCVA in treatment‐naive patients with nAMD.^[^ ^54^ ^]^ HIF‐1α inhibition restricts the countertherapeutic elevation of ANGPTL4 in RPE cells, thus enabling an adequate response in laser‐induced CNV mice compared with aflibercept monotherapy.^[^ ^55^ ^]^ These combination strategies attempt to address the compensatory responses brought by anti‐VEGF therapy.^[^ ^55^, ^56^, ^57^ ^]^ In the present study, *Ubc9* ablation in MNPs not only downregulates *Vegfa* but also the expression of other pro‐angiogenic genes including *Igf1*, *Angptl4*, and *Pdgfb*, thereby helping to reduce anti‐VEGF dosage and overcome anti‐VEGF resistance.

Of note, multiple anti‐VEGF treatment might accelerate the deterioration of the functional vascular network and exacerbate hypoxia in the retina.^[^ ^55^, ^58^, ^59^ ^]^ Besides, VEGF‐Trap (Aflibercept) could increase the number of retinal MNPs in the murine OIR model, indicating the prominent role of MNPs in pathological angiogenesis.^[^ ^27^, ^60^ ^]^ In our study, hypoxia enhances *Vegfa* stability through the UBC9/FUS pathway in the accumulated MNPs. Worse still, we identified upregulation of UBC9 post‐anti‐VEGF treatment (Figure 7A,B), since VEGF‐VEGFR2 signaling suppresses the expression of UBC9 (Figure 7C; Figure S7, Supporting Information). These findings point to a vicious cycle driven by UBC9, and the combination of UBC9 inhibition and anti‐VEGF therapy reduced vascular leakage in diabetic retinas and suppressed CNV in laser‐induced models compared with monotherapies. These results support that UBC9 inhibition serves as an effective adjuvant to anti‐VEGF therapy, which lays the foundation for treating incomplete responders or lowering the dosage of anti‐VEGF.

Nonetheless, several limitations should be recognized in the current study. First, emerging evidence has unveiled the SUMOylation‐irrelevant role of UBC9 in chromosomal rearrangement and DNA repair.^[^ ^61^, ^62^, ^63^ ^]^ Although we explored the UBC9‐mediated FUS SUMOylation in great detail, alternative mechanisms may exist. Moreover, the possible interplay between SUMOylation and other post‐translational modifications on FUS could not be excluded. Second, other pro‐angiogenic factors, like IGF1, ANGPTL4, and PDGF‐B, might also be regulated by UBC9 and contribute to the interaction between MNPs and endothelial cells. Last, as we mainly focused on pathological angiogenesis, the STZ injection, CNV, and OIR models were applied in our study. To generalize our findings in a broader patient cohort, STZ injection combined with high‐fat diet (HFD) feeding that mimics T2D‐related retinal vasculopathy could be considered for future investigations (**Figure** **8**).

## 4 Experimental Section

### Study Design

Considering the dynamic and context‐dependent effects of SUMOylation on macrophage plasticity, it was aimed to elucidate the role of SUMOylation in retinal MNPs and develop a therapeutic regimen to mitigate retinal vascular diseases. The upregulation of UBC9 in MNPs during retinal vasculopathy was verified, and that the detrimental effect of UBC9 was largely attributed to the crosstalk between MNPs and endothelial cells. MNPs‐derived cytokines related to retinopathy were screened and VEGFA was identified as a potent mediator. The mechanism was further investigated by which SUMOylation regulates VEGFA levels. It was demonstrated that the decay of *Vegfa* mRNA was controlled by the molecular switch of FUS SUMOylation. Additionally, a combination strategy with Ubc9 inhibition and anti‐VEGF therapy showed promising prospects. In the animal experiments, maximized the use of littermates was maximized and randomly assigned them to different experimental groups. The sample size was determined based on previous research. The sample size (n) for each experimental group is indicated in the Figure and Figure legends.

### Human Samples

Human blood samples were collected from ophthalmologic patients admitted to the Tongji Hospital (No.TJ‐IRB20230334). Samples were classified into the PDR group (*n* = 17) and the control group (*n* = 12) according to the medical records (Table S4, Supporting Information). PBMCs were extracted from the blood samples through gradient centrifugation with Ficoll solution (cytiva, 17 144 002). Monocytes in the cell suspension were magnetically labeled with CD11b microbeads (Miltenyi, 130‐049‐601) and retained on the LS column (Miltenyi, 130‐042‐401). Magnetically labeled monocytes were eluted and total RNA was extracted via the TRIzol (Takara, 9109) protocol. Human samples of the posterior eye segment were obtained from the Body (Organ) Donation Register and Corneal Receiving Station of Tongji Hospital of Wuhan Red Cross. The samples were divided into a diabetic group (*n* = 4) and a control group (*n* = 5) according to the donation information. An overview of the basic information can be found in Table S5 (Supporting Information). The study was approved by the Ethics Committee of Tongji Hospital, Tongji Medical College of Huazhong University of Science and Technology (No.TJ‐C20230301).

### Mouse Model

All mice were maintained in the Tongji Hospital Animal Center and protocols were approved by the Animal Research Institute Committee of Tongji Medical Center (NO.TJH‐202304001). Myeloid‐specific Ubc9 CKO mice, namely Lyzm‐Cre^+^Ubc9^fl/fl^ mice, were generated as previously described and age/sex‐matched littermates (Lyzm‐Cre^−^Ubc9^fl/fl^) were used as wild‐type control.^[^ ^13^, ^44^ ^]^ To induce diabetic retinopathy, mice were given 100 mg kg^−1^ STZ (Sigma Aldrich, S0130) intraperitoneal injection, and control mice were injected with 0.1 mm sodium citrate buffer. Three days after injection, mice with blood glucose above 16.7 mmol L^−1^ were regarded as an establishment of diabetes and were kept for 5 months until sacrifice. To induce choroidal neovascularization, mice were anesthetized with tribromoethanol, and pupils were dilated with Tropicamide/Phenylephrine Hydrochloride compound eye drops (Santen, Mydrin‐P). Bruch's membrane was ruptured at five positions with ≈72° regular intervals of the posterior pole with a 532 nm diode laser photocoagulation (ZEISS). The parameters were set as 100‐µm spot size, 100‐ms pulse interval, and 200‐mW energy. Eyes with retinal hemorrhage or lesion spot displacement were excluded for further analysis. To construct an oxygen‐induced retinopathy model, postnatal day 7 pups were maintained in a 75% ± 2% O_2_ container for consecutive 5 days, and then transferred to room air. For intravitreal injection, mice were anesthetized and pupils were dilated. Drugs (1.5 µL) were administered with a micro‐syringe (HAMILTON). For combined therapy, a total of 1.5 µL combined drugs were used.

### Integrated Analysis of scRNA‐Seq Datasets

A comprehensive bioinformatics analysis of a publicly accessible single‐cell RNA sequencing (scRNA‐seq) dataset was performed (EGAS00001004561, GSE165784).^[^ ^64^, ^65^ ^]^ Physiological retinal MG (from 2 donors) and retinal MG from PDR‐FVM (5 samples) were extracted from the downstream expression matrix. Data filtration was performed to exclude abnormal cells and genes. Preprocessing including shifted logarithm transformation, highly variable gene identification, scaling, and Leiden clustering was performed before MG population extraction. Batch effects were corrected by BBKNN in the integrated data.

### Cell Culture

BMDMs were induced as previously described.^[^ ^13^ ^]^ In brief, bone marrow cells were extracted and cultured in RPMI‐1640 (Gibco, 72 400 047) containing 10% Fetal bovine serum (Cegrogen Biotech, A0500‐3010), 1% penicillin/streptomycin (Servicebio, G4003) and 30 ng mL^−1^ M‐CSF (Biolegend, 576 408). BMDMs were polarized as previously described.^[^ ^44^ ^]^ bEnd.3 endothelial cells were obtained from Pricella (#CL‐0598, CRL‐2299). HMC3 (#ZQ0887, CVCL_II76) and HRMECs (#ZQ0884, ACBRI_181) were purchased from Shanghai Zhong Qiao Xin Zhou Biotechnology.^[^ ^66^ ^]^ HEK‐293 cells were obtained from BFB (#BFN60700191, CVCL_0063). Cells were treated with 10 µg mL^−1^ AGE‐BSA (Bioss, bs‐1158P), 1 µm Thapsigargin (MCE, 67526‐95‐8), specified concentrations of rhVEGFA (Peprotech, 100–20) and 5 µm VEGFR2 inhibitor SU5408 (MCE, 15966‐93‐5) where indicated. For tube forming assays, the ratio of BMDM‐bEnd.3 or HMC3‐HRMECs is 1:4. The plasmids (Vector, FUS‐WT, HA‐FUS‐K327R, HA‐FUS‐K502R, FUS‐2KR) were constructed by Augct Biotechnology. Additionally, other plasmids (His‐SUMO1, Flag‐UBC9) were generously provided by Li Xing^[^ ^14^ ^]^ and Xie Hao.^[^ ^67^ ^]^ The EGFP‐labeled adenoviruses (Vector, FUS‐WT, FUS‐2KR) were packaged by ObiO Technology.

### Immunofluorescence Microscopy

For vascular permeability assay, 50 mg kg^−1^ FITC‐dextran (Sigma Aldrich, FD40) was injected through the tail vein. After 20 min, mice were sacrificed via overdose intraperitoneal injection of 1.25% Tribromoethanol (Meilunbio, MA0478). Eyes were enucleated and subjected to 4% paraformaldehyde fixation for 15 min and 2× PBS for 15 min. Cup shaped retina was dissected and mounted on adhesive glass slides. One retina from each mouse was viewed and analyzed under a scanning confocal microscope (Nikon). The primary antibodies included anti‐IBA1 (Abcam, ab178846), anti‐UBC9 (Santa Cruz, sc‐271057), anti‐CD31 (Abcam, ab222783), and anti‐F4/80 (Santa Cruz, sc‐377009). Paraffin sections of human and mouse eyeball tissues were stained with primary antibodies and subsequent secondary antibodies. Imaging was performed on the biological microscope (Olympus). For retina mount immunofluorescent staining, indicated antibodies were applied and finally viewed under high‐resolution confocal microscopy (Zeiss). For vessel density quantification, the angiotool^[^ ^68^ ^]^ was applied to measure the capillaries. The retina whole mount scanning was viewed and evaluated via the caseviewer (3DHISTECH).

### Flow Cytometric Staining

After euthanasia, retinas were dissected from the eyes of diabetic mice in ice‐bath DMEM. Retinas were dissociated in DMEM supplemented with 20 U mL^−1^ Dispase II (Sigma, D4693) and 0.1 mg mL^−1^ DNase I (Roche, 10 104 159 001) for 20 min in a 37 °C water bath. The dissociated tissues were filtered by a 70 µm cell strainer (Biosharp, BS‐70‐XBS). Antibodies (FITC anti‐mouse CX3CR1 (Biolegend, 149 019), PE anti‐mouse CD11b (Biolegend, 101 208), and APC anti‐mouse CD45 (Biolegend, 103 112) were employed for the staining of the single cell suspensions. For gating, cells were also stained with the corresponding isotype antibodies. Data acquisition was performed on flow cytometers (Miltenyi MACS Quant), and the analysis was performed with FlowJo.

### Antibody Array Assay

The cytokine array assay was performed according to the manufacturer's protocol (Abcam, ab211069). In brief, BMDM samples were prepared in cell lysis buffer. The concentration of protein was quantified via BCA regents (Servicebio, G2026) before the assay. The arrayed chip membranes were incubated with samples and then antibody cocktails. The membranes were incubated with labeled HRP‐streptavidin and viewed by chemiluminescence detection. Signal density was analyzed and compared by ImageJ (NIH).

### ELISA Assay

The cultured medium of BMDMs was collected. VEGFA in the supernatant was measured by the m‐VEGFA ELISA kit (DAKEWE, 1 217 342). The VEGFA levels were quantified by comparing to the cytokine standard curve.

### Matrigel‐Plug Assay

The Matrigel‐plug assay was performed according to a previously described protocol.^[^ ^31^ ^]^ Specifically, Liquid Matrigel (Corning, 356 234) was mixed with human‐165 recombinant protein (Peprotech, 100–20). The final concentration of dissolved rhVEGFA is 0.5 µg mL^−1^. Matrigel mix (0.5 mL) was injected subcutaneously into the abdomen of 8‐week‐old C57BL/6 mice. Mice were sacrificed one week after the injection. The plug was carefully isolated from the surrounding tissues. RNA was extracted for subsequent RT‐qPCR analysis. The mRNA expression levels of target genes were normalized to the levels of mβ‐actin only.

### Transwell Assay

BMDMs were differentiated in the lower chamber of a Transwell plate (Corning, costar3422). For the Transwell assay, the previous medium was discarded and 600 µL fresh medium (10%FBS) was supplemented to the lower chamber. Approximately 20 000/well bEnd.3 cells were seeded evenly in the upper chamber. Two separate plates were kept in a 37 °C normoxic (20% O_2_) or hypoxic chamber (1% O_2_) for 16 h. The migrated cells from each filter were fixed with 4% paraformaldehyde and stained with 0.05% crystal violet. Cells that remained on the upper surface were cleared. The Transwell chamber was then observed under a microscope (Leica). The cell numbers were determined by ImageJ. This protocol is applicable to the co‐culture of HMC3 cells and HRMECs.

### Tube/Cord Formation Assay

To evaluate the angiogenic potential of endothelial cells, the in vitro tube formation assay was performed. Differentiated BMDMs or HMC3 (5000 cells well^−1^) and bEnd.3 or HRMEC (20 000 cells well^−1^) were co‐seeded in 96‐well culture plates precoated with Matrigel Matrix (Corning, 356 234). Two separate plates were kept in a 37 °C normoxic (20% O_2_) or hypoxic chamber (1% O_2_) for 6 h. The vascular network was observed under a microscope (Leica). The formed tubes and branched points were quantified using ImageJ.

### Western Blot Analysis

The cells were washed twice with cold 1× PBS and lysed in RIPA lysis buffer (Beyotime, P0013B) containing 1% protease inhibitor cocktail (Abclonal, RM02916) and 1% phosphatase inhibitor cocktail (Servicebio, G2007) for 30 min in an ice bath. A BCA kit (Servicebio, G2026) was used to measure the protein concentration. The SDS PAGE loading buffer (Beyotime, P0286) was added to the cell lysate and heated in a 95 °C water bath for 6–8 min. Approximately 20 µg samples were loaded onto the SDS PAGE gel and run for 1 h 30 min to 2 h. The proteins were transferred to a 0.45 µm PVDF membrane (Millipore, IPVH00010) bathed in cold transfer buffer (Glycine, Tris‐Base, ddH_2_O, and methanol). The membranes were blocked with 5% skim milk (Biosharp, BS102) for 1 h at room temperature and incubated with the indicated primary antibodies (1:500–1:1000) diluted in enhanced antibody dilution buffer on a shaker at 4 °C overnight. After 5 × 10 min washes with TBST, membranes were incubated with HRP‐conjugated secondary antibodies for 1 h at room temperature and for another 5 washes. After 2 min of incubation of the enhanced chemiluminescent solution, the membranes were exposed through an X‐ray film or a GelView imaging system (BLT). The relative intensities of the bands were analyzed and normalized to the indicated internal control using ImageJ. The following primary antibodies were used: anti‐UBC9 (CST, 4786), anti‐ACTB (Abclonal, AC038), anti‐VEGFA(A12303), anti‐ZO‐1(Abclonal, A0659), anti‐Occludin (Proteintech, 27260‐1‐AP), anti‐VEGFR2 (Affinity, AF6281), anti‐Phospho‐VEGFR2 (Affinity, AF4426), anti‐AKT (CST, 9272), anti‐Phospho‐AKT (CST, 4060), anti‐SUMO1 (CST, 4940), anti‐FUS (Abclonal, A21830), anti‐HA (CST, 3724), anti‐FLAG (CST, 2368), anti‐HIS (CST, 2366), anti‐GAPDH (Abclonal, AC001).

### Co‐Immunoprecipitation Assay

Cells were washed twice with cold 4 °C 1× PBS and collected in IP lysis buffer (Beyotime, P0013) with 10 mm NEM (Sigma Aldrich, 0 4260), 1% protease inhibitor cocktail (Abclonal, RM02916), 1% phosphatase inhibitor cocktail (Servicebio, G2007), and 1% PMSF (Meilunbio, MA0001). Sonication at 40% power for 2 cycles and centrifugation at 12 000 g for 10 min were performed, followed by collection of the supernatant. The lysate was pre‐cleared by incubation with Dynabeads (Invitrogen, 1004d) on an orbital shaker for 2 h at 4 °C, and the input lysates were separated. The supernatant was then incubated with the primary antibody or control IgG and beads overnight at 4 °C on an orbital shaker. The beads were washed five times in 1× PBS buffer to remove unbound proteins. The protein complex was eluted by incubation with lysis buffer on an ice bath for 30 min and heated (95 °C) with loading buffer. The beads were removed then and prepared for WB analysis.

### IP‐MS Analysis

BMDMs differentiated from WT and Ubc9 CKO mice were subject to hypoxic conditions and then lysed by IP lysis buffer (Beyotime, P0013) containing 1% protease inhibitor cocktail (Abclonal, RM02916), 1% phosphatase Inhibitor (Servicebio, G2007), 1 mm PMSF (MeilunBio, MA0001) and 10 mm N‐ethylmaleimide (NEM) (Sigma, 0 4260). The anti‐SUMO1 antibody (CST, 4940) was used for immunoprecipitation. After the western blot, the gel underwent coomassie brilliant blue staining. Then the gel was subjected to liquid chromatography with tandem mass spectrometry for proteomic analysis (Jingjie PTM BioLab).

### RNA Stability Assay

BMDMs were treated as indicated in the figure legends. Actinomycin D (MCE, HY‐17559) was added to a final concentration of 10 µg mL^−1^ in the culture medium. Cell samples were collected at 0, 1, and 2 h following Actinomycin D treatment. RNA was extracted via the TRIzol protocol. For quantification, β‐actin served as an internal control for RT‐qPCR.

### RT‐qPCR Assay

RNA extraction was performed using TRIzol reagent (Takara). After the removal of genomic DNA with 4 × gDNA Wiper (Vazyme, R223), the products were subjected to reverse transcription with 5× HiscriptII Qrt SuperMix II. For the pre‐mRNA analysis, a kit with random hexamers was used (Vazyme, R212). The cDNA from various cell samples was then amplified by real‐time qPCR with the indicated primers using the LightCycler 96 Instrument (Roche). The transcriptional level of genes was calculated using the 2*^−ΔΔCt^* method and normalized to β‐actin. The primers are listed in Table S2 (Supporting Information).

### siRNA, Plasmid and Adenovirus Transfection/Transduction

Transfection of siRNA into HMC3 cells was performed with Lipofectamine 3000 (Thermo Fisher Scientific, L3000015) according to the manufacturer's protocol. Lipofectamine RNAiMAX Transfection Reagent (Thermo Fisher Scientific, 13 778 150) was used to transfect siRNA into BMDMs. To introduce plasmid DNA into HEK‐293T cells, HighGene plus Transfection Reagent was used (Abclonal, RM09014P). Adenoviruses were transduced to BMDMs after the elimination of endogenous FUS through the 3′UTR‐targeted siRNA. A preliminary test was established to explore the appropriate MOI for BMDM transduction. After siRNA transfection (for 12 h), the previous medium was replaced with a fresh culture medium (FBS free) containing the indicated adenoviruses. Two hours later, fresh culture medium (10%FBS) was added to the well and 12 h later the medium was replaced. After 36 h, subsequent experiments were performed.

### RNA Immunoprecipitation and RNA Pull‐down

BMDMs were transduced with indicated adenoviruses (FUS‐WT, FUS‐2KR). An RNA‐binding Protein immunoprecipitation Kit (Bersinbio, Bes5101) was applied. The anti‐HA antibody (CST, 3724) was used and co‐precipitated RNAs were detected by RT‐qPCR. For RNA pull‐down, a biotin‐labeled oligo (flanking on 3′UTR AU‐rich element of *Vegfa*) was constructed and the experiment was performed by using an RNA pull‐down Kit (Bersinbio, Bes5102). Briefly, protein lysates were incubated with the biotin‐labeled probe to form the RNA‐protein complex. Streptavidin‐labeled magnetic beads were added to probe this complex. After elution, a Western blot was employed to detect the protein portion.

### Preparation of siRNA‐Loaded Liposome

The preparation of siRNA‐loaded liposomes was carried out according to the previously reported method.^[^ ^69^, ^70^ ^]^ Anhydrous ethanol was used to dissolve Lipoid, cholesterol, DSPC, and mPEG‐DMG in a molar ratio of 50:38.5:10:1.5. siRNA was dissolved in citrate buffer (10 mm, pH = 3). Lipid solutions were supplemented to siRNA solutions during high velocity vortex (≥2000 rpm) and then subjected to 10 kD ultrafiltration tubes (Merck). After centrifugation (3000 g), the siRNA‐loaded liposomes were dissolved in 1× PBS.

### Optical Coherence Tomography Angiography

Before in vivo imaging, mice pupils were dilated. To ensure the image quality, the anesthetized mouse was immobilized on an operating platform positioned adjacent to the scanning lens. Microvascular imaging was performed using a full‐range swept‐source OCT device (TowardPi). The en face OCTA images were analyzed.

### Statistical Analysis

The number of replicates within the experiment is indicated in the Figure and Figure legends. Comparisons between two groups were performed using the unpaired student's *t*‐test and one‐way ANOVA was used for comparing three or more groups. Statistical analysis of the data was conducted using the GraphPad Prism 9 software (GraphPad Software Inc.). All results were expressed as mean ± SEM. *p*‐value < 0.05 was considered statistically significant.

## Conflict of Interest

The authors declare no conflict of interest.

## Author Contributions

Z.Z., H.M.Y., J.Q.L., and Z.Q.Z. collected human samples. Z.Z., G.Y.L., R.H.W., and X.H.M. carried out the experiments and analyzed the data. Z.Z. and G.Y.L. wrote the manuscript. Z.Z. and G.Y.L. contribute evenly to this project.

## Supporting information



Supporting Information

## Data Availability

The data that support the findings of this study are available from the corresponding author upon reasonable request.
