# Supplementary material for: SUMOylation is a Translatable Target in Hypoxic MNPs Regulating Retinal Vasculopathy
Source: Adv Sci (Weinh). 2025 May 31;12(31):e03505. doi: 10.1002/advs.202503505 (PMC12376520; doi:10.1002/advs.202503505)
Supplement: Supplementary file 1 — Supporting Information [file ADVS-12-e03505-s001.docx]

**Supplemental Information**


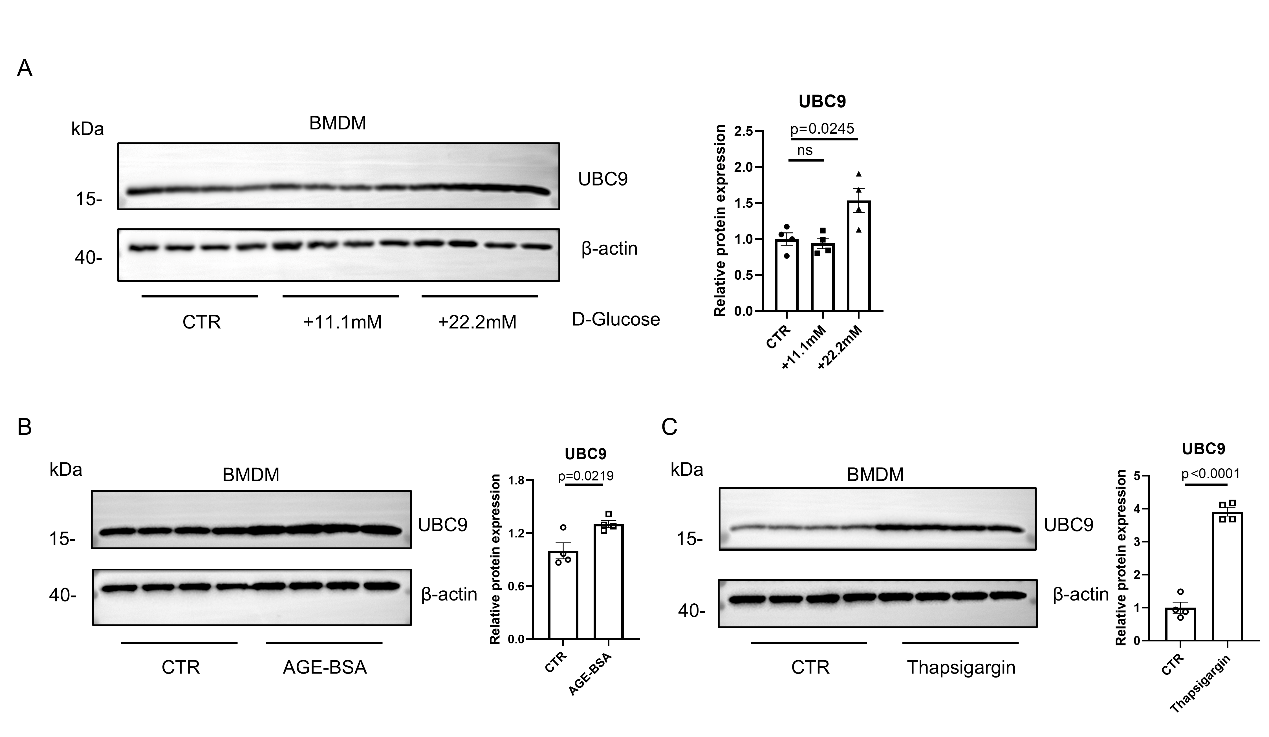


Figure S1. UBC9 is upregulated in macrophages upon glucose and AGE stimulation as well as ER stress induction.

(A-C) BMDMs were stimulated with (A) varied concentrations of glucose (high glucose: 22.2mM), (B) BSA-conjugated advanced glycosylation end-products (AGE-BSA), and (C) the ER stress inducer, Thapsigargin. Expression of UBC9 was determined by Western blot (n=4).

Unpaired two-tailed Student’s t test was used for statistical analysis. Data are presented as mean ± SEM.


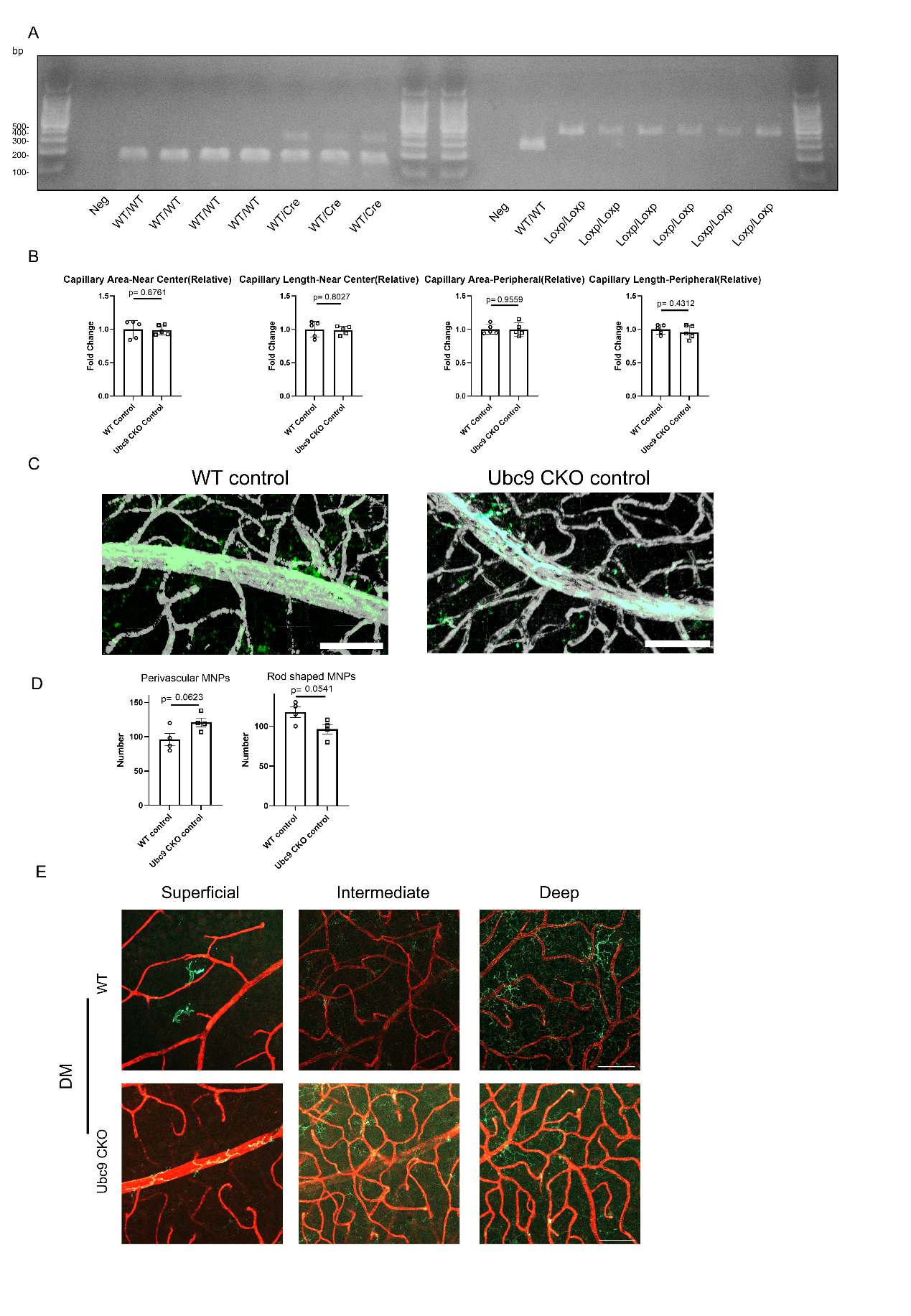


Figure S2. The effect of conditional UBC9 ablation on the morphology and spatial distribution of retinal MNPs in normal control mice.

A) Representative gel image for mouse genotyping.

B) Quantification of retinal capillaries in normal control mice (n=5).

C) 3D-rendered imaging of the retinal MNPs (F4/80^+^, green) and retinal vasculature (CD31^+^, grey) in normal control mice (scale bar=50μm).

D) Perivascular MNPs and rod-shaped MNPs were counted through retina flat-mount scanning (n=4).

E) Representative images of retinal MNPs (F4/80^+^, green) and retinal vasculature (CD31^+^, red) within the superficial, intermediate and deep layers of diabetic retina (scale bar=50μm).

Unpaired two-tailed Student’s t test was used for statistical analysis. Data are presented as mean ± SEM.


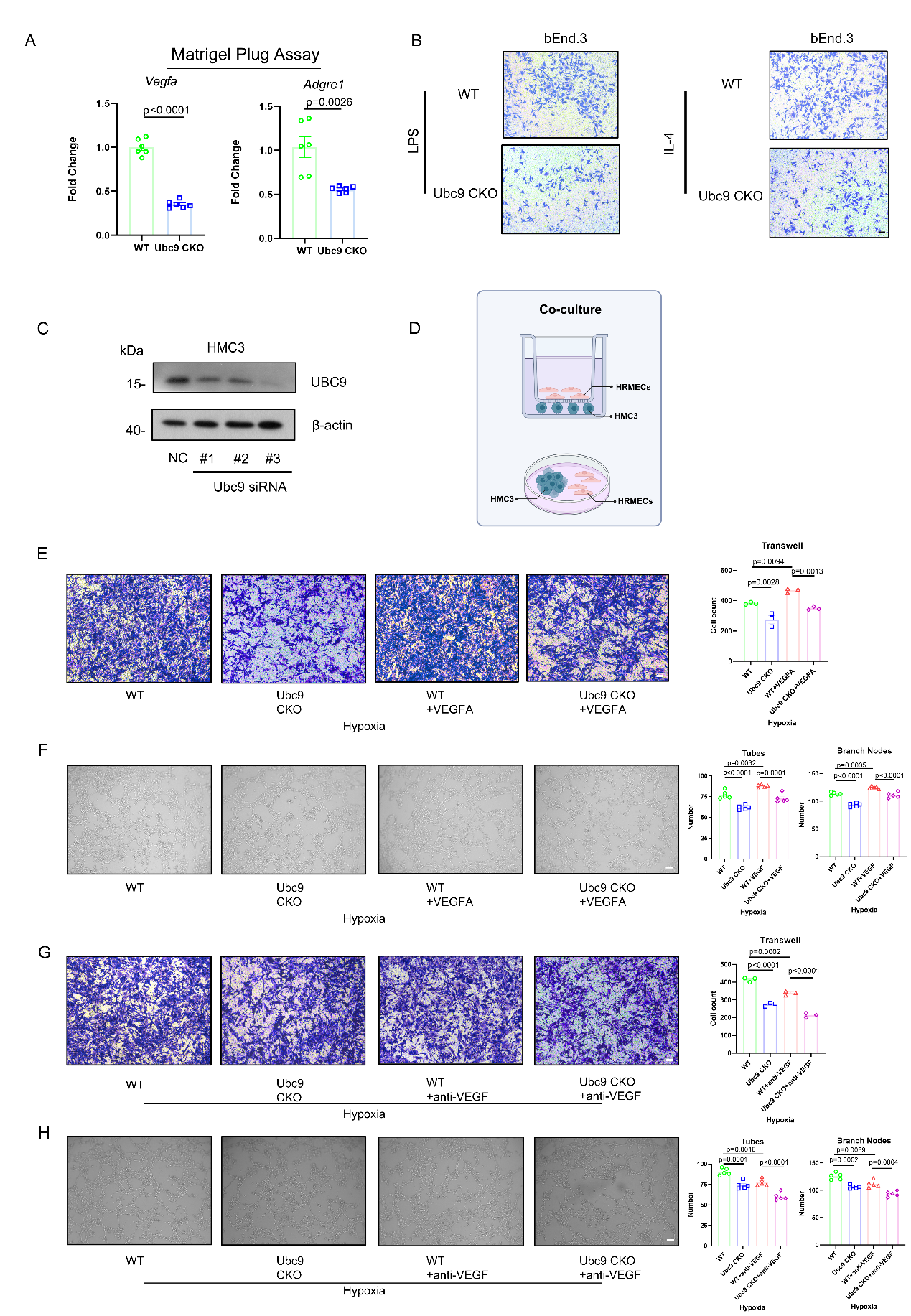


Figure S3. UBC9 modulates the pro-angiogenic capacity of macrophages.

A) Levels of *Vegfa* and *Adgre1* transcripts detected by RT-qPCR in the subcutaneous Matrigel plugs (n=6).

B) Representative image of the migratory bEnd.3 cells attracted by LPS or IL-4 stimulated BMDMs.

C) Protein levels of UBC9 after siRNA#1, #2 and #3 treatment in HMC3 cells.

D) Schematic representation of the co-culture of HMC3 and HRMECs.

E) Transwell analysis of bEnd.3 cells co-cultured with hypoxic BMDMs (scale=20μm), and 50ng/ml VEGFA was supplemented in indicated groups (n=3).

F) Representative images of bEnd.3 cells and hypoxic BMDMs co-seeded in Matrigel (scale=20μm), and 50ng/ml VEGFA was supplemented in indicated groups (n=5).

G) Transwell analysis of bEnd.3 cells co-cultured with hypoxic BMDMs (scale=20μm), and 100μg/ml ranibizumab (anti-VEGF) was applied in indicated groups (n=3).

H) Representative images of bEnd.3 cells and hypoxic BMDMs co-seeded in Matrigel (scale=20μm), and 100μg/ml ranibizumab (anti-VEGFA) was applied in indicated groups (n=5).

Unpaired two-tailed Student’s t test or one way ANOVA with Bonferroni’s multiple comparisons test was used for statistical analysis. Data are presented as the mean ± SEM.


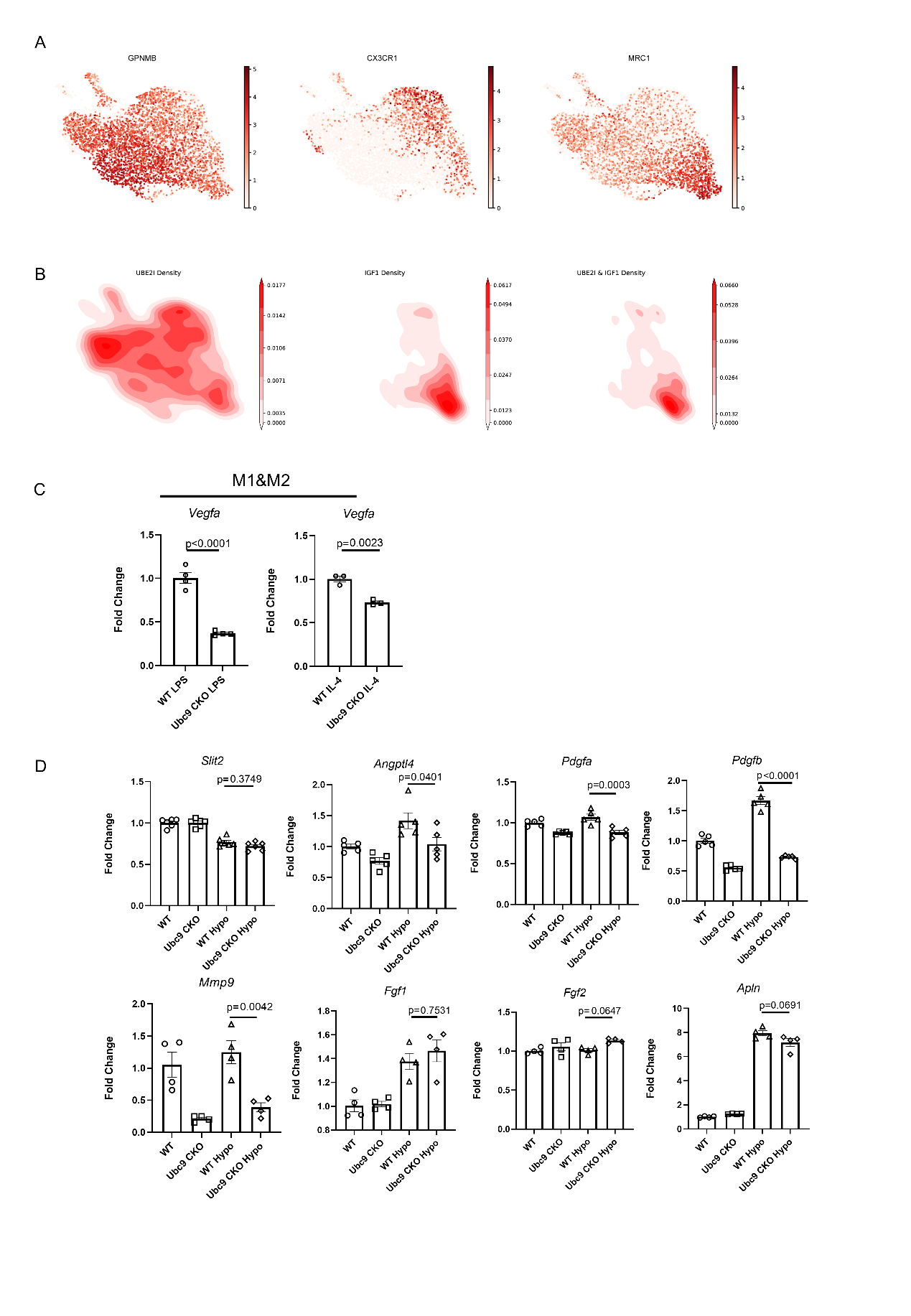


Figure S4. UBC9 regulates the expression of various pro-angiogenic factors in addition to VEGFA.

A) Cell markers (GPNMB, CX3CR1, MRC1) for the identification of microglia subpopulations.

B) Weighted density plot showing the (co-)expression of UBE2I and IGF1 within the retinal microglia subpopulations.

C) Expression levels of *Vegfa* transcripts in BMDMs after LPS (M1, n=4) or IL-4 (M2, n=3) treatment.

D) Expression levels of *Slit2* (n=5), *Angptl4* (n=5), *Pdgfa* (n=5), *Pdgfb* (n=5), *Mmp9* (n=4), *Fgf1* (n=4), *Fgf2* (n=4) and *Apln* (n=4) transcripts in WT and Ubc9 CKO BMDMs cultured under normoxic and hypoxic conditions.

Unpaired two-tailed Student’s t test or one way ANOVA with Bonferroni’s multiple comparisons test was used for statistical analysis. Data are presented as the mean ± SEM.


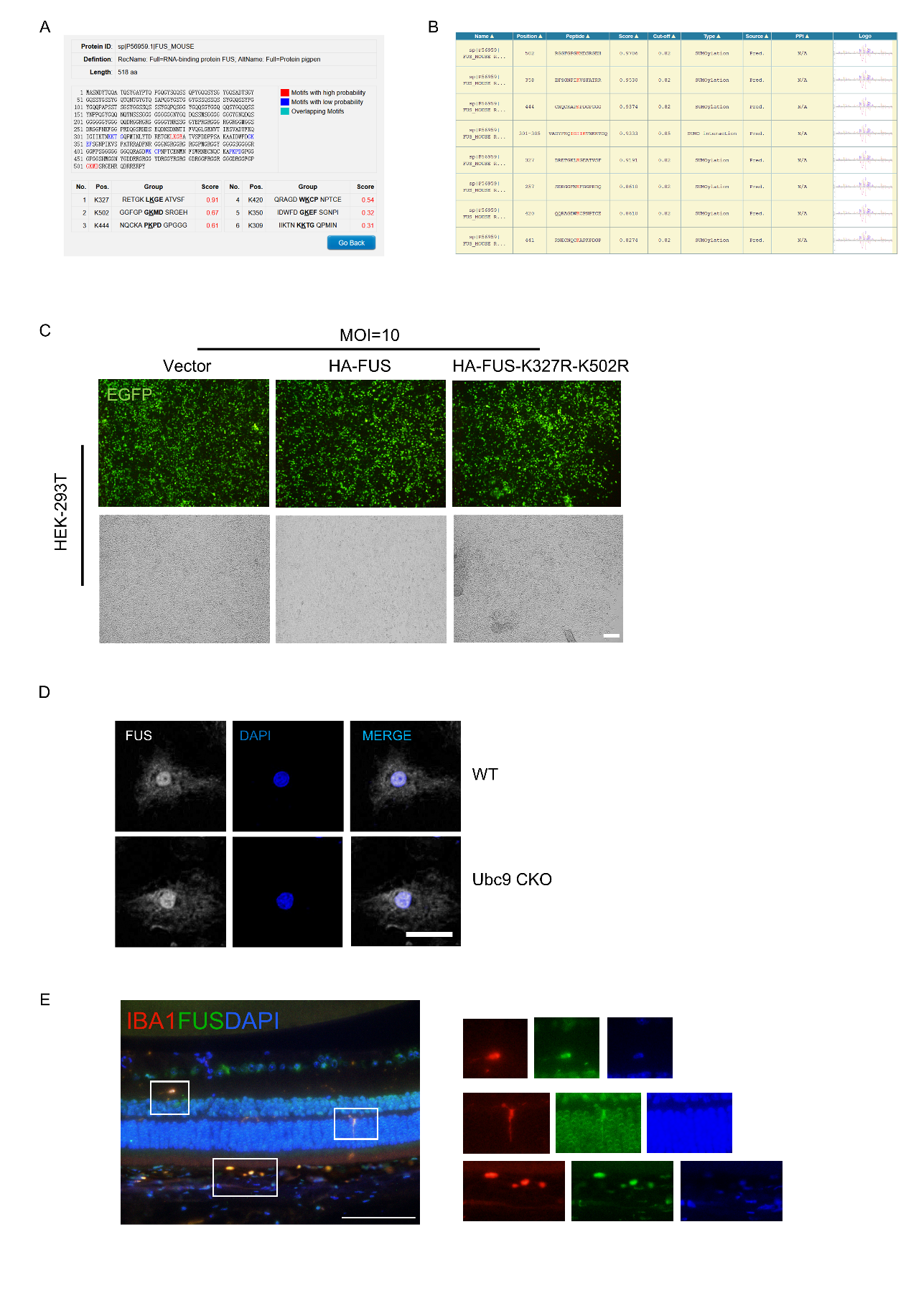
Figure S5. Prediction of potential SUMOylation sites in FUS.

1. SUMOylation site prediction using SUMOplot Analysis.
2. SUMOylation site prediction using GPS-SUMO Analysis.
3. Adenovirus transduction at the indicated MOI in HEK-293T cells (scale bar=200μm).
4. Subcellular distribution of FUS in BMDM upon UBC9 depletion (scale bar=20μm).
5. Representative immunofluorescence co-staining of FUS and IBA1 in mouse retina/choroid (scale bar=100μm).


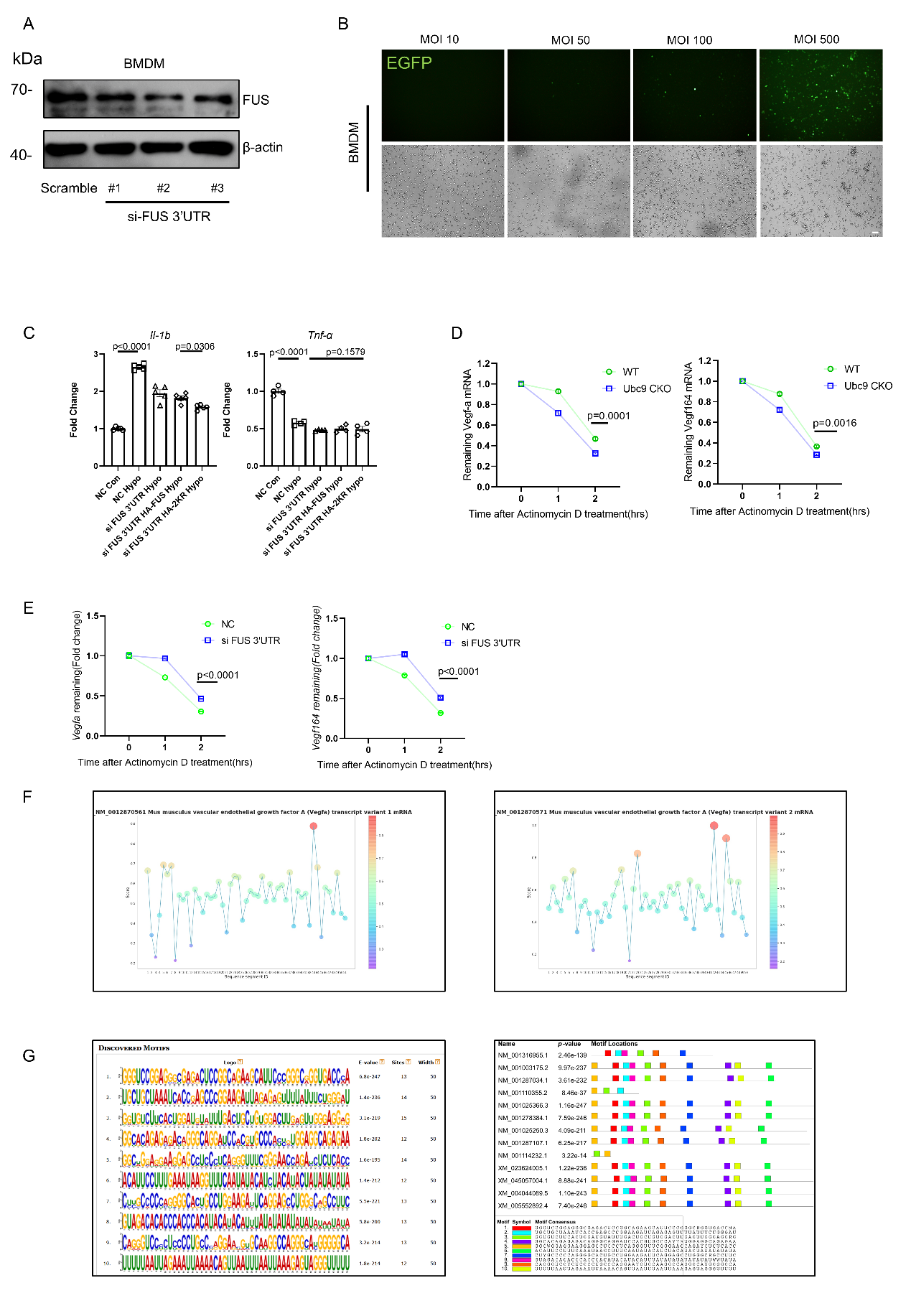


Figure S6. SUMOylation of FUS affects its binding to *Vegfa* mRNA.

1. Verification of the knockdown efficacy of FUS siRNA (on 3’UTR) in BMDMs.
2. Adenovirus transduction at the indicated MOI in BMDMs (scale bar=100μm).
3. Expression levels of *Il-1b* (n=5) and *Tnf-α* transcripts (n=4) in indicated groups.
4. Stability of *Vegfa* and *Vegf164* mRNA was measured in WT and Ubc9 CKO BMDMs after treatment with Actinomycin D (n=4).
5. Stability of *Vegfa* and *Vegf164* mRNA was measured in NC and siFUS 3’UTR BMDMs after treatment with Actinomycin D (n=8).

F-G) Prediction of FUS binding sites in *Vegfa* mRNA with RBPsuite (F). The *Vegfa* 3’UTR conservation analysis with MEME suite (G).

Unpaired two-tailed Student’s t test or one way ANOVA with Bonferroni’s multiple comparisons test was used for statistical analysis. Data are presented as the mean ± SEM.


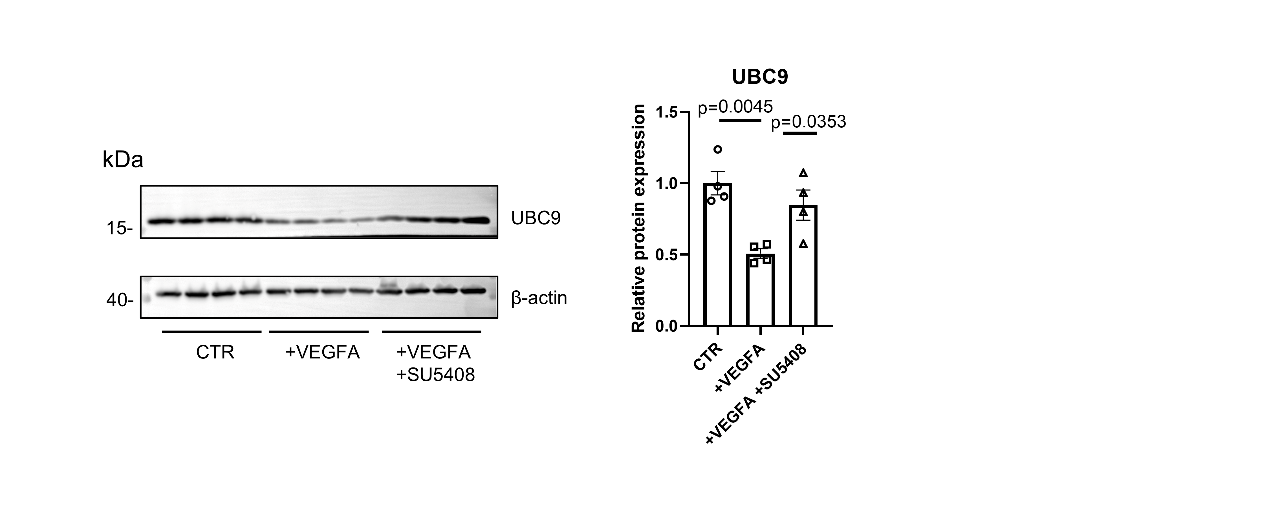


Figure S7. VEGFA down-regulates UBC9 through VEGFR2 signaling.

Expression levels of UBC9 in control (CTR), VEGFA or VEGFA+SU5408 (VEGFR2 inhibitor) treated BMDMs (n=4).

One way ANOVA with Bonferroni’s multiple comparisons test was used for statistical analysis. Data are presented as mean ± SEM.


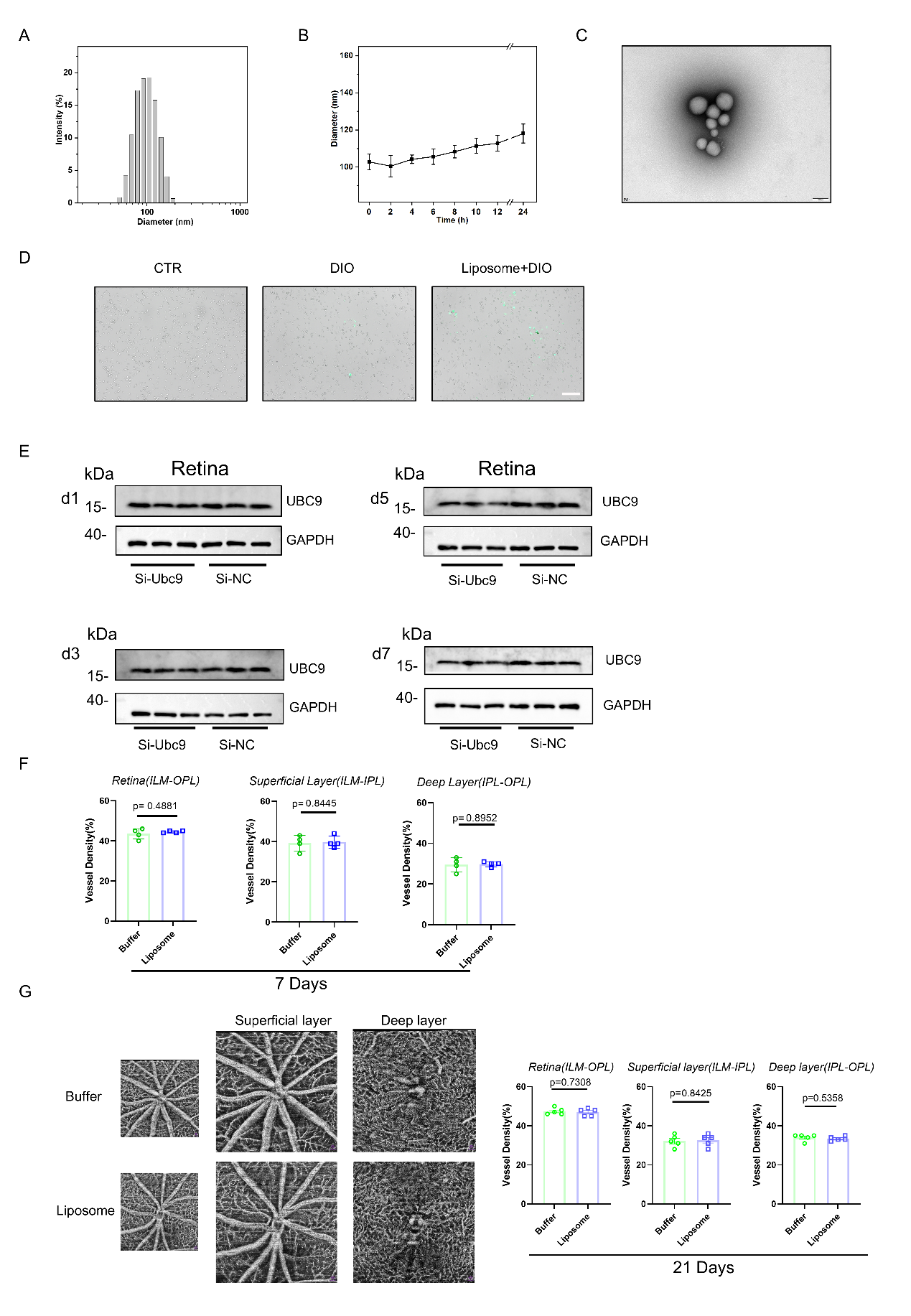


Figure S8. Delivery of siRNA-loaded liposomes.

A-C) Diameter distribution and colloid stability of siRNA-loaded liposomes.

D) Transfection of liposome-encapsulated DIO into BMDMs (scale bar=200μm).

E) Expression of UBC9 in mouse retina after intravitreal injection of Ubc9siRNA#liposome (n=3).

F) Liposome safety evaluated by vessel density alteration based on OCTA analysis 7 days after intravitreal injection (n=4).

G) Liposome safety evaluated by vessel density alteration based on OCTA analysis 21 days after intravitreal injection (n=5).

Unpaired two-tailed Student’s t test was used for statistical analysis. Data are presented as the mean ± SEM.

**Table S1**

**Primer, siRNA and oligo.**

| **Primer** |  |  |
| --- | --- | --- |
| m-Lyzm Type -co | GACCCAGCCTCCAGTCACC | |
| m-Lyzm Type -wt | AGCTGGAGCCATCAAGAAGAG | |
| m-Lyzm Type -mut | GGCCCAAATGTTGCTGGAT | |
| m-Ubc9Type_F | TACAGTGCCCACCACCACCATT | |
| m-Ubc9Type_R | CTCAGGATTCCAGCACCACACG | |
| h-*SAE1*-F | AGGACTGACCATGCTGGATCAC | |
| h-*SAE1*-R | CTCAGTGTCCACCTTCACATCC | |
| h-*UBE2I*-F | ATCCAAGACCCAGCTCAAGCAG | |
| h-*UBE2I*-R | TTGACGATGCCACAAGGTCGCT | |
| h-*RANBP2*-F | CCAGTATCTTCTCAGACCAGCAG | |
| h-*RANBP2*-R | GTGTGCATCTGTTGCGGATAGG | |
| h-*SENP1*-F | CATTTCGCCTGACCATTACACGC | |
| h-*SENP1*-R | CACACTTGGCAAGCCCTTCTCT | |
| m-*Pecam-1*-F | CCAAAGCCAGTAGCATCATGGTC | |
| m-*Pecam-1*-R | GGATGGTGAAGTTGGCTACAGG | |
| m-*Cdh5*-F | GAACGAGGACAGCAACTTCACC | |
| m-*Cdh5*-R | GTTAGCGTGCTGGTTCCAGTCA | |
| m-*Ptprc*-F | CTTCAGTGGTCCCATTGTGGTG | |
| m-*Ptprc*-R | TCAGACACCTCTGTCGCCTTAG | |
| m-*Itgam-*F | TACTTCGGGCAGTCTCTGAGTG | |
| m-*Itgam*-R | ATGGTTGCCTCCAGTCTCAGCA | |
| m-*Cspg4*-F | GAGGTCTTGGTGAACTTCACCC | |
| m-*Cspg4*-R | GACAGTAGGAGACCGATGGTGT | |
| m-*Ccl7*-F | CAGAAGGATCACCAGTAGTCGG | |
| m-*Ccl7*-R | ATAGCCTCCTCGACCCACTTCT | |
| m-*Vegfa*-F | CTGCTGTAACGATGAAGCCCTG | |
| m-*Vegfa*-R | GCTGTAGGAAGCTCATCTCTCC | |
| m-*Mmp9*-F | GCTGACTACGATAAGGACGGCA | |
| m-*Mmp9*-R | TAGTGGTGCAGGCAGAGTAGGA | |
| m-*Il-1b*-F | TGGACCTTCCAGGATGAGGACA | |
| m-*Il-1b*-R | GTTCATCTCGGAGCCTGTAGTG | |
| m-*Tnfα*-F | GGTGCCTATGTCTCAGCCTCTT | |
| m-*Tnfα*-R | GCCATAGAACTGATGAGAGGGAG | |
| m-premRNA-*Vegfa*-F | TTTGACCTAGAGGCTGCTCC | |
| m-premRNA-*Vegfa*-R | AGGCTCCAAGGAATTAGACAGC | |
| m-*Vegf120*-F | GAAGCTACTGCCGTCCGATT | |
| m-*Vegf120*-R | GCCTTGGCTTGTCACATTTTTC | |
| m-*Vegf144*-F | GAAGTCCCATGAAGTGATCAAG | |
| m-*Vegf144*-R | TCGGCTTGTCACATACGCTC | |
| m-*Vegf164*-F | GTCCGATTGAGACCCTGGTG | |
| m-*Vegf164*-R | CAAGGCTCACAGTGATTTTCTGG | |
| m-*Vegf188*-F | AAGCTACTGCCGTCCGATTG | |
| m-*Vegf188*-R | AAGGCTCACAGTGAACGCTCC | |
| m-*Vegfa*-3’UTR-1-F | TACGGTACTTATTTAATAGCCCTT | |
| m-*Vegfa*-3’UTR-1-R | CAGGACAAGCTAGTGACTGT | |
| m-*Vegfa*-3’UTR-2-F | ACGGTACTTATTTAATAGCCCTT | |
| m-*Vegfa*-3’UTR-2-R | GAGAGCGAGAGATACATCTCATAA | |
| m-*Vegfa*-3’UTR-3-F | TCATTTATTTATTGGTGCTACTGTT | |
| m-*Vegfa*-3’UTR-3-R | TGCTCTAGAGACAAAGACGTG | |
| m-*β-actin*-F | CATTGCTGACAGGATGCAGAAGG | |
| m-*β-actin*-R | TGCTGGAAGGTGGACAGTGAGG | |
| h-*β-actin*-F | CACCATTGGCAATGAGCGGTTC | |
| h-*β-actin*-R | AGGTCTTTGCGGATGTCCACGT | |
| m-*F4/80(Adgre1)*-F | CGTGTTGTTGGTGGCACTGTGA | |
| m-*F4/80(Adgre1)*-R | CCACATCAGTGTTCCAGGAGAC | |
| m-*Slit2*-F | ACCGTCTGAGATGTATCCCTCC | |
| m-*Slit2*-R | GCTGACAAGTCATTGAAGGCACC | |
| m-*Angptl4*-F | CTGGACAGTGATTCAGAGACGC | |
| m-*Angptl4*-R | GATGCTGTGCATCTTTTCCAGGC | |
| m-*Pdgfa*-F | CTGGCTCGAAGTCAGATCCACA | |
| m-*Pdgfa*-R | GACTTGTCTCCAAGGCATCCTC | |
| m-*Pdgfb*-F | AATGCTGAGCGACCACTCCATC | |
| m-*Pdgfb*-R | TCGGGTCATGTTCAAGTCCAGC | |
| m-*Fgf1*-F | CCAAGGAAACGTCCACAGTCAG | |
| m-*Fgf1*-R | ACGGCTGAAGACATCCTGTCTC | |
| m-*Fgf2*-F | AAGCGGCTCTACTGCAAGAACG | |
| m-*Fgf2*-R | CCTTGATAGACACAACTCCTCTC | |
| m-*Apln*-F | AGGCATAGCGTCCTCACCTCTT | |
| m-*Apln*-R | GGTGCAGAAACGACAAAGACGG | |
| **siRNA** |  | |
| h-si -Ubc9-1-F | CAAAGAUGAUUAUCCAUCU TT | |
| h-si -Ubc9-1-R | AGAUGGAUAAUCAUCUUUG TT | |
| h-si -Ubc9-2-F | UCACAAUCAAACAGAUCCU TT | |
| h-si -Ubc9-2-R | AGGAUCUGUUUGAUUGUGA TT | |
| h-si -Ubc9-3-F | GCACAAGCCAAGAAGUUUG TT | |
| h-si -Ubc9-3-R | CAAACUUCUUGGCUUGUGC TT | |
| m-si -Ubc9-1-F | AUACACGUUUGGAUGAAACAG TT | |
| m-si -Ubc9-1-R | CUGUUUCAUCCAAACGUGUAU TT | |
| m-si -Ubc9-2-F | AUUUAGAAGUUCUUGUAUUCC TT | |
| m-si -Ubc9-2-R | GGAAUACAAGAACUUCUAAAU TT | |
| m-si -Ubc9-3-F | UCUUGUAUUCCUAAUAAGAUC TT | |
| m-si -Ubc9-3-R | GAUCUUAUUAGGAAUACAAGA TT | |
| m-si-Fus-3’UTR-1-F | GGUAGUCUGACACACACACAC TT | |
| m-si-Fus-3’UTR-1-R | GUGUGUGUGUGUCAGACUACC TT | |
| m-si-Fus-3’UTR-2-F | GGUAACACUGGGUACAGGAAG TT | |
| m-si-Fus-3’UTR-2-R | CUUCCUGUACCCAGUGUUACC TT | |
| m-si-Fus-3’UTR-3-F | UCUGACACACACACACACACA TT | |
| m-si-Fus-3’UTR-3-R | UGUGUGUGUGUGUGUGUCAGA TT | |
| m-si-NC-3’UTR-1-F | UUCUCCGAACGUGUCACGU TT | |
| m-si-NC-3’UTR-1-R | ACGUGACACGUUCGGAGAA TT | |
| m-si-NC-3’UTR-2-F | UUCUCCGAACGUGUCACGU TT | |
| m-si-NC-3’UTR-2-R | ACGUGACACGUUCGGAGAA TT | |
| h-si-NC-F | UUCUCCGAACGUGUCACGU TT | |
| h-si-NC-R | ACGUGACACGUUCGGAGAA TT | |
| **Oligo** |  | |
| PullDown-3’UTR-AU-probe | Bio-UUUUAAUUAGAAAUUAAAACAGUUAAUUUAAUUAAAGAGUAGGGUUUUUUUCAGUAUUCUUGGUUAAUAUUUA | |
| NC-probe | Bio-UUUUUUUUAGAAAAAAAAACAGUUUUUUUUUUUUUAGAGAAGGGUUUUUUUCAGUUUUCUUGGAAAAAAAAAA | |

**Table S2**

Other resources

| Software | Source | Identifier |
| --- | --- | --- |
| Prism version 9.0.0 | Graphpad | https://www.graphpad.com/ |
| Image J 1.52v | NIH | https://imagej.net/Welcome |
| Zen 3.8(blue edition) | Zeiss | https://www.zeiss.com/microscopy/zh/products/software/zeiss-zen.html |
| Caseviewer2.3 | 3DHISTECH | https://www.3dhistech.com/solutions/caseviewer/ |
| FlowJo (V10) | TreeStar | https://www.flowjo.com/solutions/flowjo/downloads |
| Python | Python Software Foundation | https://www.python.org/ |

**Table S3**

Characterization of patients (blood sample donors)

| Case | Race | Age | Gender | Diagnosis | Diabetes or not | Diabetes duration(years) | HbA1c (%[mmol/mol]) | Fasting Blood Glucose (mmol/l) | Other systemic disease | Insulin usage/other medication for diabetes | Anti-VEGF treatment history | Ophthalmologic notes |
| --- | --- | --- | --- | --- | --- | --- | --- | --- | --- | --- | --- | --- |
| 1 | East Asian | 40 | male | Retinal Detachment | Non-diabetes | - | - | 5.36 | - | - | - | Complicated cataract |
| 2 | East Asian | 56 | female | Complicated cataract | Non-diabetes | - | - | 4.8 | Hypertension | - | - | Angle-Closure Glaucoma |
| 3 | East Asian | 54 | male | Retinal Detachment | Non-diabetes | - | - | 5.09 | - | - | - | High myopia |
| 4 | East Asian | 59 | female | Retinal Detachment | Non-diabetes | - | - | 5.05 | - | - | - | Cataract |
| 5 | East Asian | 88 | female | Complicated cataract | Non-diabetes | - | - | 5.63 | - | - | - | Pterygium |
| 6 | East Asian | 59 | male | Vitreous Hemorrhage (Ocular contusion) | Non-diabetes | - | - | 5.21 | - | - | - | Traumatic Cataract |
| 7 | East Asian | 66 | female | Vitreous Hemorrhage (Ocular contusion) | Non-diabetes | - | - | 5.10 | - | - | - | Complicated cataract |
| 8 | East Asian | 73 | female | Idiopathic Macular Hole | Non-diabetes | - | - | 5.64 | - | - | - | Complicated cataract |
| 9 | East Asian | 51 | male | Complicated cataract | Non-diabetes | - | - | 4.65 | Chronic Hepatitis B | - | - | High Myopia |
| 10 | East Asian | 81 | male | Complicated cataract | Non-diabetes | - | - | 6.02 | - | - | - | High Myopia  Macular Hole |
| 11 | East Asian | 63 | female | Macular Epiretinal Membrane | Non-diabetes | - | - | 5.3 | Hypertension | - | - | - |
| 12 | East Asian | 73 | female | Complicated cataract | Non-diabetes | - | - | 4.62 | - | - | - | Primary angle-closure glaucoma |
| 13 | East Asian | 65 | female | Vitreous Hemorrhage | Diabetes | 20+ | N/A | 4.75 | Coronary Heart Disease | - | - | Diabetic macular oedema |
| 14 | East Asian | 60 | male | Vitreous Hemorrhage | Diabetes | - | 7.0 | 5.12 | - | N/A | N/A | Diabetic macular oedema |
| 15 | East Asian | 52 | male | Vitreous Hemorrhage | Diabetes | 14 | 10 | 7.56 | Diabetic Nephropathy, Hypertension,  Chronic Heart Failure | Insulin | N/A | Diabetic macular oedema, complicated cataract |
| 16 | East Asian | 30 | male | Vitreous Hemorrhage | Diabetes | 1 | N/A | 9 | Hypertension,  Ankylosing Spondylitis | Insulin | - | Diabetic macular oedema |
| 17 | East Asian | 61 | female | Vitreous Hemorrhage | Diabetes | N/A | N/A | 4.33 | Coronary Heart Disease | Acarbose | +(1) | Diabetic macular oedema, Complicated cataract |
| 18 | East Asian | 68 | male | Vitreous Hemorrhage | Diabetes | N/A | 7.9 | 8.18 | - | N/A | N/A | Diabetic macular oedema |
| 19 | East Asian | 41 | male | Vitreous Hemorrhage | Diabetes | 15 | 5.3 | 4.16 | Hypertension,  End-stage Renal Disease | Nateglinide | +(3) | Diabetic macular oedema |
| 20 | East Asian | 37 | male | Vitreous Hemorrhage | Diabetes | 12 | 9.3 | 7.44 | - | Insulin | +(1) | Diabetic macular oedema, Iris neovascularization |
| 21 | East Asian | 60 | female | Vitreous Hemorrhage | Diabetes | 11+ | 5.4 | 4.62 | Hypertension, Cerebral infarction | Glimepiride | +(N/A) | Diabetic macular oedema |
| 22 | East Asian | 54 | female | Vitreous Hemorrhage | Diabetes | 10+ | N/A | 4.60 | - | N/A | - | Diabetic macular oedema, Glaucoma |
| 23 | East Asian | 59 | male | Vitreous Hemorrhage | Diabetes | 20+ | 6.9 | 6.33 | Hypertension, Hypothyroidism, Coronary Heart Disease | Insulin | - | Diabetic macular oedema |
| 24 | East Asian | 77 | female | Vitreous Hemorrhage | Diabetes | 30+ | 7.4 | 8.39 | Chronic renal failure | Insulin | - | Complicated cataract, Diabetic macular oedema |
| 25 | East Asian | 50 | male | Vitreous Hemorrhage | Diabetes | 20+ | 9.2 | 12.14 | Diabetic Nephropathy | Insulin/Metformin/ Dapagliflozin | - | Complicated cataract, Diabetic macular oedema |
| 26 | East Asian | 52 | female | Vitreous Hemorrhage | Diabetes | 5 | 8.7 | 8.18 | Hypertension, Diabetic Nephropathy, Diabetic Peripheral Neuropathy | Metformin/ Repaglinide/ Acarbose | - | Complicated cataract, Diabetic macular oedema |
| 27 | East Asian | 58 | female | Diabetic Tractional Retinal Detachment | Diabetes | 10 | 9.8 | 5.25 | - | - | N/A | Vitreous Hemorrhage |
| 28 | East Asian | 40 | male | Vitreous Hemorrhage | Diabetes | 8 | 7.6 | 5.96 | - | Insulin | N/A | Diabetic macular oedema |
| 29 | East Asian | 57 | male | Vitreous Hemorrhage | Diabetes | 20 | 10.2 | 12.14 | Hypertension | Insulin | - | Complicated cataract, Diabetic macular oedema |

**TableS4**

Characterization of ocular posterior segment donors

| Case | RACE | Age(death) | Gender | Cause of death | Diabetes or not | Eye(s) |
| --- | --- | --- | --- | --- | --- | --- |
| 1 | East asian | 65 | male | N/A | non-diabetes | OD |
| 2 | East asian | 52 | male | N/A | non-diabetes | OS |
| 3 | East asian | 61 | female | Leukemia | non-diabetes | OD |
| 4 | East asian | 42 | male | Craniocerebral injury | non-diabetes | OS |
| 5 | East asian | 46 | male | Craniocerebral injury&respiratory failure | non-diabetes | OS |
| 6 | East asian | 44 | male | Respiratory failure & diabetic ketoacidosis (cadio-respiratory arrest) | diabetes | OD |
| 7 | East asian | 75 | male | Respiratory failure | diabetes | OD |
| 8 | East asian | 50 | male | Brainstem hemorrhage-Cardiopulmonary resuscitation-respiratory failure | diabetes | OD |
| 9 | East asian | 65 | male | Kidney failure | diabetes | OS |

Table S5

BioRender License

| NO. | Agreement Number |
| --- | --- |
| 1. | BW287E4H8D |
| 2. | MB287E477N |
| 3. | KL287E4IMW |
| 4. | FV287E49B0 |
| 5. | HW287E488C |
| 6. | MQ287E48B7 |
| 7. | OQ287E4A65 |
| 8. | EV287E4M9Q |
